# Fatty Acid Content and Profile in *Ulva lactuca* in Response to Exposure to Variable Growth Conditions in Indoor Photobioreactors

**DOI:** 10.3390/life15010057

**Published:** 2025-01-06

**Authors:** Nabeel Gnayem, Razan Unis, Rima Gnaim, Alexander Chemodanov, Álvaro Israel, Jallal Gnaim, Alexander Golberg

**Affiliations:** 1 Department of Environmental Studies, Porter School of Environment and Earth Sciences, Tel Aviv University, Tel Aviv-Yafo 6997801, Israel; 2 The Triangle Regional Research and Development Center, Kfar Qari 3007500, Israel; 3 Israel Oceanographic and Limnological Research Institute, Haifa 3109701, Israel

**Keywords:** *Ulva lactuca*, growing conditions, indoor photobioreactor, fatty acid, PUFA, omega-3, omega-6, seaweed, macroalgae

## Abstract

Seaweed presents a sustainable alternative source of valuable fatty acids (FAs) involving omega-3 (*n*-3) and omega-6 (*n-6*). As such, there is great potential to reduce pressure on wild fish populations, helping to combat overfishing and its associated global impacts. This study explored the effect of various environmental factors on the FA content and profile of *Ulva lactuca* using indoor photobioreactors. The taxonomic identity of *U. lactuca* was confirmed through DNA sequencing using 3 markers (rbcL, ITS, and tufa). The effects of temperature (8, 20, and 30 °C), seawater salinity (3.5, 3.0, 2.5, and 2.0% *w/v*), nutrient type and concentration (0 or 6.4 ppm, consisting of 50% *w/w* N-NO_3_, 50% *w/w* N-NH_4_, and 0–1 ppm P-PO_4_), and irradiance (50, 100, and 150 μmol photons m^−2^ s^−1^) were evaluated. This study assessed their influence on *U. lactuca*’s biomass production rate (BPR), dry weight (DW), ash content (AC), and FA composition after 7 and 21 days. The results revealed that after 21 days, the polyunsaturated FA (PUFA) content decreased with the increasing seawater salinity (i.e., 38.9% ± 0.7, 33.8% ± 0.4, and 27.0% ± 0.4, and 6.6% ± 0.1 for a salinity of 2.0, 2.5, 3.0, and 3.5% *w/v*, respectively). The content of *n-3* after 21 days increased significantly under the following conditions: 8 °C, a salinity of 2.5% *w/v*, 6.4 ppm of nitrogen without the addition of phosphorous, and an irradiation of 50 and 150 μmol photons m^−2^ s^−1^, affording a low *n-6/n-3* proportion that fits a desirable level of an *n6/n3* ratio (1–10) for a balanced nutritional diet.

## 1. Introduction

Marine macroalgae (seaweed) are multicellular, photoautotrophic organisms that rely on dissolved inorganic carbon dioxide, light, and nutrients to form the foundation of various food chains within marine ecosystems [1]. Seaweed has massive potential as feedstocks for producing a broad spectrum of biochemicals for human benefit and use [2]. Seaweed is attracting growing interest, primarily due to its offshore cultivation, eliminating the need for agricultural land use. Moreover, seaweed has a high potential as an alternative to a vegetable diet, thus augmenting the food supply chain [3]. The fast-advancing algae-based bioeconomy highlights the importance of developing sustainable produce that safeguards natural seaweed populations [4]. These biochemical compounds can be further engineered and optimized by manipulating the seaweed’s growth conditions [5]. Cultivating seaweed may also mitigate climate change and reduce environmental damage associated with fossil fuel use [6].

Based on their pigmentation, the primary phyla of seaweeds are Chlorophyta (green), Rhodophyta (red), and Ochrophyta–Phaeophyceae (brown) [1,7,8]. The Israeli Mediterranean Sea in the Levantine basin offers a unique macro-habitat in which more than 300 seaweed species can be found [9,10]. Nonetheless, the Levantine basin is strongly oligotrophic, and two significant events have strongly influenced the environmental character and marine life on the Israeli Mediterranean shores [9]. The inauguration of the Suez Canal enabled the migration of marine organisms, such as seaweeds, from the Red Sea to the Mediterranean; unfortunately, the arrival of invasive herbivorous fish reduced the biodiversity and biomass of seaweeds [11]. Second, the Aswan Dam was established in the 1960s, consequently tempering the flow of the Nile and lowering the amount of freshwater and minerals arriving in the Levantine basin [9]. The Red Sea biodiversity revealed the presence of 133 species for the *Phylum* Chlorophyta, 157 species for the *Phylum* Ochrophyta, and 286 species for Rhodophyta [11,12,13].

A unique potential of marine green algal biorefineries lies in their ability to produce a diverse range of yields, for instance, food, animal feed, biofuels, added to biomaterials, while taking advantage of the open ocean for cultivation [14,15,16,17,18]. Unlike conventional agriculture, which relies heavily on irrigated crops, offshore cultivation techniques eliminate the need for additional nutrient inputs. This reliance carries substantial environmental costs, contributing significantly to carbon emissions and the eutrophication of aquatic ecosystems [3,19]. Land-based cultures (onshore) can be operated under controlled (nutrient supply) or partially regulated (light and temperature) growth variables, providing more sustainable and uniform quality algal products [18].

*Ulva* sp. is an edible green seaweed known for its rapid growth in natural environments and its remarkable ability to withstand significant abiotic fluctuations, such as changes in temperature and salinity [5]. In most studies exploring *Ulva* sp. as a biorefinery feedstock, the biomass is harvested from natural populations or cultivated in large outdoor tanks, marine environments, or small laboratory setups. Small laboratory flasks and tubes, which facilitate maintaining clean cultures, can further support precisely controlled experimental environments [20,21].

Fats play a crucial role in maintaining human health, but it is important to consume them in the right amounts and alongside other key nutrients to maintain a proper balance, such as vitamins, carbohydrates, proteins, and minerals [22]. Lipids’ primary role is to provide energy; however, they are also needed to maintain cell membrane integrity and to produce hormones. Moreover, lipids are essential for transporting and absorbing fat-soluble vitamins [23,24]. It is increasingly important to explore new food sources that minimize the strain on land-based ecosystems and help decrease their overuse [25]. As a novel raw material for the food industry, seaweeds offer a valuable source of fatty acids. Although their overall lipid content is relatively low, they are rich in essential unsaturated fatty acids (EUFAs), which are pivotal for human welfare; in particular, seaweeds synthesize *n-3* and *n-6* [1]. The lipid properties of seaweeds are species-specific, with their FA concentrations and profiles subject to variation in reaction to diverse biotic and abiotic factors [26]. Thus, there is a need to study different natural seaweed species to understand their potential for industrial application and exploitation in applications such as food products or dietary supplements [22].

Seaweeds have a lipid content varying between 0.6 and 9.0% from DW [27]; therefore, they would be regarded as a good source of UFAs [28]. *Calliblepharis jubata* (red seaweed) and *Undaria pinnatifida* (brown seaweed) possess a high content of PUFAs and highly unsaturated FAs (HUFAs), along with relatively low *n-6/n-3* ratios (of 0.01 and 0.09, respectively), which may contribute to the prevention of diseases and the improvement in the quality of life [22]. Green seaweeds have a lipid content that varies between species and is influenced by temporal conditions and the seasons [29,30,31,32].

PUFAs in algae are generally classified into two prominent families: linoleic FA (LA, *n-6*) and α-linolenic acid (ALA, *n-3*). PUFAs constitute a noteworthy component of sea algal fats, with microalgae serving as fish’s primary basis of omega FA. Among the most crucial PUFAs are the essential unsaturated FA (EUFAs) eicosapentaenoic acid (EPA, *n-3*) and docosahexaenoic acid (DHA, *n-3*) [29,33].

*Ulva* is found worldwide and has been extensively researched due to its beneficial biochemical properties, fast growth, and adaptability [34]. The lipid extract of *Ulva* species is primarily composed of palmitic, oleic, and linoleic FAs; SFAs represent around 50–80% of the entire FAs (TFAs) [30,35]. Monounsaturated fatty acids (MUFA) account for a high percentage: 17.6–33.4% *w/w* of the TFA. PUFAs are elevated in *Ulva* sp. extract, reaching a very high level in winter added to spring (38.4% *w/w* of TFA). The abundance of PUFAs is linked to the association with cooler temperatures and the potential to adjust the fatty acid composition by optimizing cultivation practices [30,35].

The *Ulva* sp. compound profile is influenced by several factors, including the specific species [36,37], the physical development site [38,39], microclimate [18,40], age added to the picking stage [41], and the season of picking [42]. Annual term variations, including variations in sunlight radiation, salinity, disposal of fertilizer elements, and seawater heat, also play a role [20]. Since most *Ulva* populations originate from shores and estuaries, with fewer found in ocean lagoons, coastal lakes, canals, and rivers, their chemical profile reflects the salinity conditions of these habitats (0.05 to 4.9% *w/v*) [34,43].

*Ulva* sp. production and biochemical profile are influenced by the airflow amount introduced into farming systems through motorized encouragement, CO_2_ enrichment, or enhanced nutrient mass transfer [44]. As the fertilization concentration increases, protein content rises, while starch content declines [44]. High irradiance (about 150 μmol m^−2^ s^−1^) and relatively low temp. (about 12 °C) were reported as optimal conditions for the growth of *Ulva fenestrata* (Chlorophyta) [45]. However, adding nutrients or changing the CO_2_ levels was unnecessary to elevate its growth. Low light levels produced the most elevated FA, protein, and phenolic levels. In contrast, low temperature decreases FA content but positively affects the protein level. Adding nutrients, particularly nitrates, enhances the levels of fatty acids (FAs), protein, and phenolic compounds [45]. Biochemical analysis of biomass cultivated at an optimized nutrient level (60 μM nitrate and 6 μM phosphate) indicated that it has 9.3% protein, 20.2% carbohydrates, and 6.3% lipids on a DW basis [46]. The growth rate of *Ulva ohnoi* increased for incubation at a salinity of 1.5, 2.5, and 3.5% *w/v* and at a phosphorus concentration of 1.0 μmol L^−1^ in aquaculture [47].

This study aimed to examine how short-term exposure, when varying the irradiance, salinity, nutrients, and temperature, affects biomass production, carbon hydrogen nitrogen sulfur and phosphorus (CHNSP) elemental content, ash content (AC), dry weight DW, FA content, and FA profile in *U. lactuca*. This study can provide valuable insights into optimizing *U. lactuca* biomass production and biochemical composition. It could also contribute to a better understanding of how environmental changes influence marine ecosystems and algal physiology.

## 2. Materials and Methods

### 2.1. Molecular Identification of Ulva sp.

These experiments used a fresh algal mass (50-day-old *U. lactuca*) of healthy, clean Talhi cultured in outdoor containers at the Israel Oceanographic and Limnological Research Institute (IOLR). A dry algal thallus (10–50 mg) sample was cleaned using sterilized pretend salt water, frozen in fluid nitrogen, and then ground. The DNA was extracted and purified from the *Ulva* sp. samples by Plant Genomic DNA Lineage Kit (Bioneer, Daedoek-gu, South Korea) as detailed by [21].

### 2.2. Salinity Experiment

**Figure 1:**
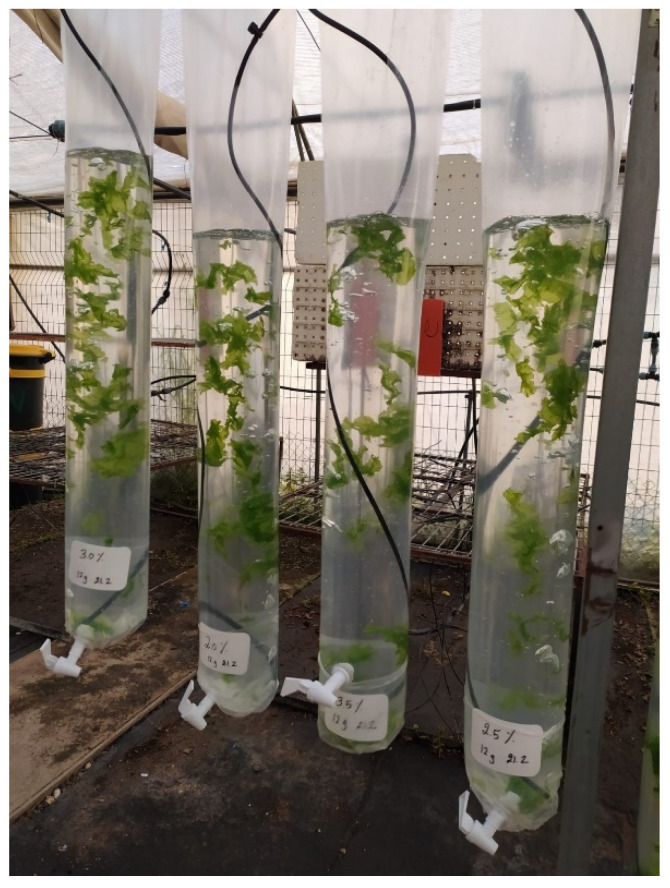
*Ulva lactuca* seaweed, cultivated in a 10 L plastic sleeve, which served as a photobioreactor, was situated in an outdoor plastic house at the Triangle Research and Development (TDRC). The cultivation conditions included an irradiance of µmol m^−2^ s^−1^, a 12/12 h light/dark photoperiod, and an artificial seawater (ASW) pH maintained at 8.2. The temperature was controlled at 25 °C during the day and 15 °C at night. A total of 12 fresh samples (3 replicates) were collected for each of the 4 treatments with ASW salinity concentrations of 2.0%, 2.5%, 3.0%, and 3.5%.

In total, 10 g of a fresh biomass starter of *U. lactuca* was rinsed with artificial seawater (ASW), then raised in an outdoor vertical plastic sleeve functioning as a marine photobioreactor (Figure 1), following the methodology outlined by [48]. The sleeve system obtained the perfect transparent and vertical cheap, affordable, and accessible method for growers and laboratories. The farming vessel was assembled using welding plastic wrappers (Genigar, Kibbutz Ginegar, Israel, with a width of 40 cm and about 0.2 mm thickness), including a UVA stabilizer, and facilitated aeration by aeration stone, totaling 10 L of ASW with 2.0, 2.5, 3.0, and 3.5% salinity. An aquarium air stone was located at the bottom, while the ASW was replaced at the upper. The aeration and water replacement were hand-regulated and used for every reactor by motorized valves. This marine photobioreactor allowed for the acclimation, cultivation, and maintenance of seaweed species under natural conditions, as described in the temperature testing system and according to [49]. Seaweed’s Biomass production ratio (BPR) was intended to be based on the entire mass bulk per cultivation day relative to the size of the growth solution (in kg m^−3^ day^−1^). The harvested biomass was subjected to color and shape inspection. Freshly collected seaweed samples (40 g, *n* = 3) were rinsed using double-distilled water (DDW), dehydrated in an oven at 105 °C, and then kept in isolated tubes at −20 °C for storage. Ash content is measured as the residue weight after burning in an oven at 550 °C.

### 2.3. Temperature Experiment

The experiment was conducted in a plastic house in Israel’s Triangle Regional R&D Center (TRDC, Kafr Qara, Israel). The system consisted of three water paths (10 L of distilled water) incubators (MRC, Holon, Israel), equipped with thermostats and a circulation system, which were set at different temperatures (8, 20, and 30 °C). The temperature levels were selected to represent three main seasons, reflecting the sea water temperature during winter, spring, and summer accordingly. Each treatment utilized three transparent plastic jars (1000 mL) with a lid. The jar system was selected to fit transparent and closed containers to include the biomass and isolate the ASW from the bath systems. The jars were filled with 800 mL substrate of artificial seawater (ASW), which was prepared by dissolving 35 g of Red Sea salt (produced by Red Sea Company, Herzliya, Israel), reaching a concentration of 35 ppt (3.5% *w/v*). Each jar was aerated using a glass pasture pipette connected to a 6 mm aquarium silicone pipe, which is connected to an air pump of Antman 35 W, 200 L h^−1^. The air enriched the water with CO_2_ and circulated the water in the jars. A fresh algal biomass (*U. lactuca* cultured in outdoor containers in the IOLR for 50 days) was used to select the starting material for these experiments. Fresh and healthy thali (1 g) were selected, washed, and added to 800 mL of culture medium in plastic jars, each treatment with triplicates (3 jars for each temperature level). The algal material was cultured in 12:12 h light/dark sequence in an incubator with different temperatures (8, 20, and 30 °C). The medium in the jars was fertilized with 6.4 ppm of N (50% of N-NH_4_ and 50% of N-NO_3_ using (NH_4_)_2_SO_4_ and Ca(NO_3_)_2_, respectively), and 1 ppm of P (H_3_PO_4_), which was added to the media once every five days. The pH in the medium was kept at 8.2. The air temperature was monitored, and a range of 7–26 °C was observed in the plastic house used as an outdoor shelter for the experiment. The natural irradiation intensity range in the greenhouse was 300–700 w m^−2^ s^−1^ at day time. These conditions of nutrients, PH, irradiation, and salinity were kept as standard to avoid variation in parameters other than the temperature. The standard levels of the growing conditions regarding fertilizers, PH, radiation, and air temperature were selected following the protocols of outdoor growing conditions in ILOR facilities of or research group. After 7 days, the thali was isolated from the media in each flask and dried by a manual salad centrifuge until no water was observed. The wet weight was recorded for each replicate (*n* = 3), and samples (optimized in a preliminary testes to ensure significancy) of 1 g were taken from each flask to determine the biomass DW, AC, elemental analysis, and FA profiling of *U. lactuca.* The biomass was dried in paper bags in the oven at 105 °C for 48 h and kept at −80 °C in sealed plastic bags.

### 2.4. Irradiation Experiment

This experiment was conducted in the TRDC laboratory to determine how the irradiation strength (50, 100, or 150 µmol m^−2^ s^−1^ PAR), illuminated by an LED lamp (the spectrum is presented in Figure S1), affected fresh *U. lactuca* inside 1 L flasks filled with 800 mL of artificial seawater (3.5% red sea salt). The levels of irradiation were selected based on various preliminary tests. Flasks that were aerated and fertilized. The temperature and pH were determined as described in the temperature experiment. One gram of fresh and healthy thali was harvested, washed, and added to 800 mL of culture medium in 9 flasks (three flasks for each irradiation level). The algal material was cultured using a 12:12 h light/dark rotation in an incubator with different irradiation levels (50, 100, or 150). Freshly harvested samples were dried after 7 and 21 days and kept at −80 °C in sealed plastic bags.

### 2.5. Nutrient Experiment

The experiment was conducted in the TRDC laboratory to investigate the effect of nitrogen and phosphorus nutrients using nitrogen at 0 or 6.4 ppm and phosphorus at 0 or 1 ppm. Four treatments were tested: N0P0, N6.4P1, N0P1, and N6.4P0. The selected levels of fertilizers follow the protocols of growing conditions in the research group’s facilities. Fresh *U. lactuca* was placed inside 1 L flasks (12 flasks, 3 replicates for each of the four treatments) and filled with 800 mL of ASW (3.5%). An LED bar with 100 m^−2^ s^−1^ was used under the same conditions (temperature, aeration, pH, and photoperiod) as detailed in the irradiation experiment.

### 2.6. Biomass Collection and Saving

Fresh thali (*n* = 3) was collected from each treatment group following cultivation periods of 7 and 21 days using the method outlined by [35].

### 2.7. DW, AC, and Elemental Analysis

The weighed samples of fresh *U. lactuca* from all experiments were dried [50,51], and the DW, AC, and elemental analysis were determined as described by [35].

### 2.8. Directly Methylation of FA Followed by Investigation Using Gas Chromatography-Mass Spectrometry (GC-MS)

In total, 25 mg of dehydrated algal fine powder was mixed with methanol and dichloromethane (2 + 1 mL accordingly), sulfuric acid (0.3 mL, 10% *v/v*), and internal standard C19:0 (20 µg, from Sigma, Berlin, Germany, 98% purity) in an intense-walled glass heaviness tube (5 mL)-added agitator magnate. The tube was sealed securely and heated to 90 °C for 18 h. After cooling to room temp., the blend was neutralized to pH 7 using a 20% *w/v* NaOH solution and transferred into a hexane–water blend (ratio of 2:1 *v/v*, 5 mL). The supernatant organic level was rinsed with 3 mL of double distilled water, sorted out by filter, and scaled after evaporating the diluter (38). The remains were dissolved in 1 mL of high pure hexane and passed through a siring and micro-filter. The FA methyl esters (FAMEs) in the supernatant were investigated by GC-MS (Thermo Trace 1310 GC), equipped with a capillary column (TG-SQC GC) and a mass spectrometer detector (ISQ LT), as (35) outlined. GC-MS (Thermo Trace 1310 GC, Waltham, MA USA) with software NIST08 libraries (Mainlib, NIST-MSMS, NIST-MSMS2, NIST-RI, and Replib) can identify tens of thousands of molecules. After separating the fatty acids in the GC, each fatty acid molecule reaches the mass spectrometer (MS). The MS (ISQ LT) ionizes the molecules, producing charged fragments with specific mass-to-charge (*m/z*) ratios. The resulting mass spectra represent a unique “fingerprint” for each fatty acid. The mass spectra obtained from the sample were compared with all reference libraries (comprehensive databases of mass spectra) of known fatty acids to provide a similarity score (e.g., percentage match) between the unknown fatty acids and the library entries. The fatty acids in the sample were identified based on the best matches and the corresponding similarity scores. Usually, a higher similarity score indicates a more confident identification. The analyzed fatty acid was quantified based on the response factor compared to that of the internal standard C19:0 used in the GC-MS analysis.

### 2.9. Statistical Analysis

Each measurement was performed three times, and the outcomes showed an average of replicate ± standard deviation (SD). A statistical examination was conducted using JMP-IN software version 5.0.1a (SAS Institute, Inc., Cary, NC, USA) and ANOVA one-way using Tukey HSD. This analysis revealed significative differences among the samples.

## 3. Results

### 3.1. Identification of the Ulva Species DNA Marker

DNA barcodes were acquired for all experiments to start stocking fresh biomass. Samples were identified using the ITS region, which ranged from 400 to 500 bp, and the rbcL region, which spanned 1000 to 1300 bp. DNA sequences of algal experiments matched the *U. lactuca* series with the corresponding concurrence numbers EU933990.1 and MK167980.1 and contributed to 98–100% parallels [21,35,52].

### 3.2. Effect of Salinity

**Figure 2:**
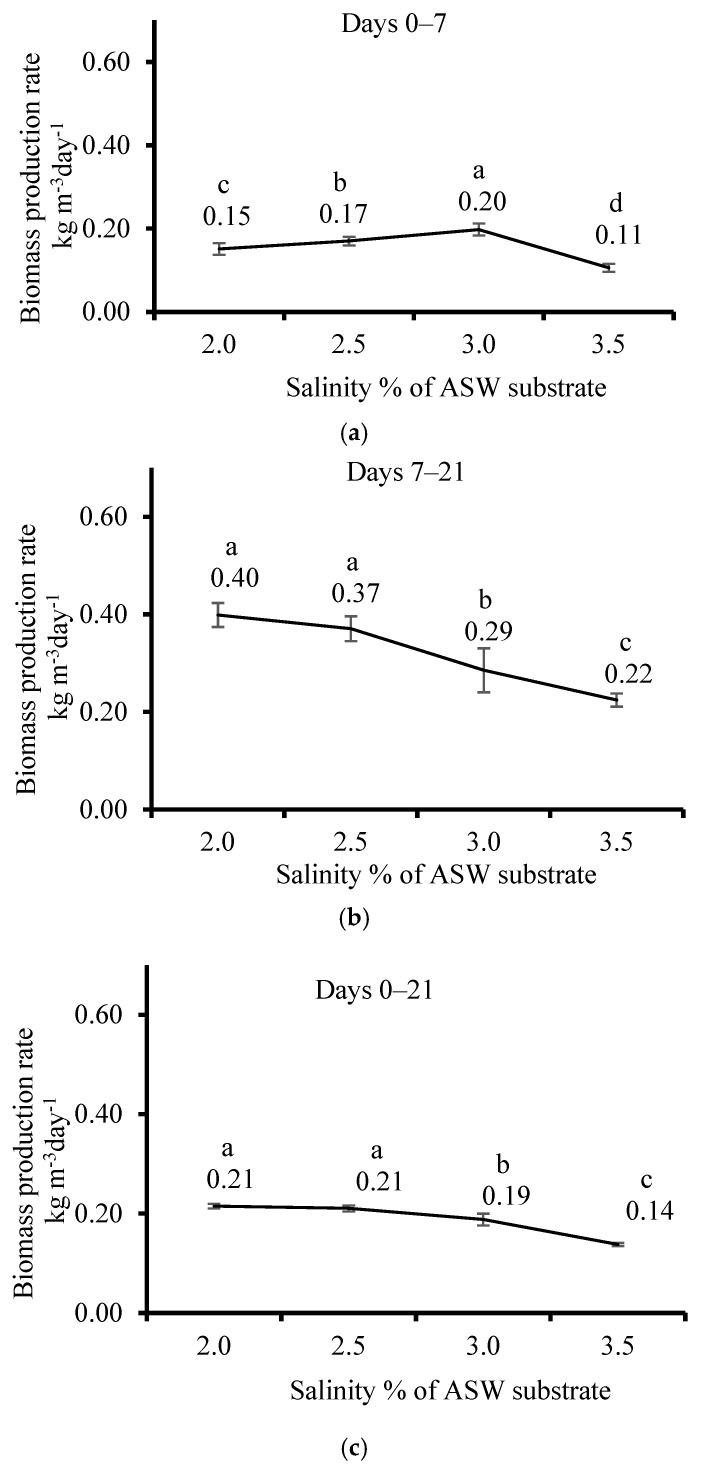
The effect of salinity in the artificial seawater (ASW) substrate with *Ulva lactuca* cultivated in a sleeve system outdoors in a plastic house on the biomass production rate (BPR, kg m^−3^ day^−1^, mean ± SD, *n* = 3) was measured after (**a**) 0–7 days, (**b**) 8–21 days, and (**c**) 0–21 days. Different letters above the values indicate significant differences as determined by the one-way ANOVA Tukey HSD test (*p* < 0.05).

The thali of the *U. lactuca* shape became shrunken and curled. Their color darkened (Figure S2) when exposed to 2.0% salinity, compared with the typical flat shape and color standard green, when exposed to 2.5, 3.0, and 3.5% salinity of ASW. The BPR during the first 7 days of cultivation (Figure 2a) showed significant differences between the BPR of different salinity levels: 3.0% was the highest, followed by 2.5%, 2.0%, and 3.5%, which was the minimal (0.2, 0.15, 0.17, and 0.11 kg m^−3^ day^−1^, accordingly). BPR decreased significantly with increased salinity, both after 7 days (Figure 2b) (0.4, 0.37, 0.29, and 0.22 kg m^−3^ day ^−1^ for 2.0, 2.5, 3.0, and 3.5%, respectively), and after 21 days (Figure 2c) (0.21, 0.21, 0.19, and 0.4 kg m^−3^ day^−1^ for 2.0, 2.5, 3.0, and 3.5%, respectively).

**Figure 3:**
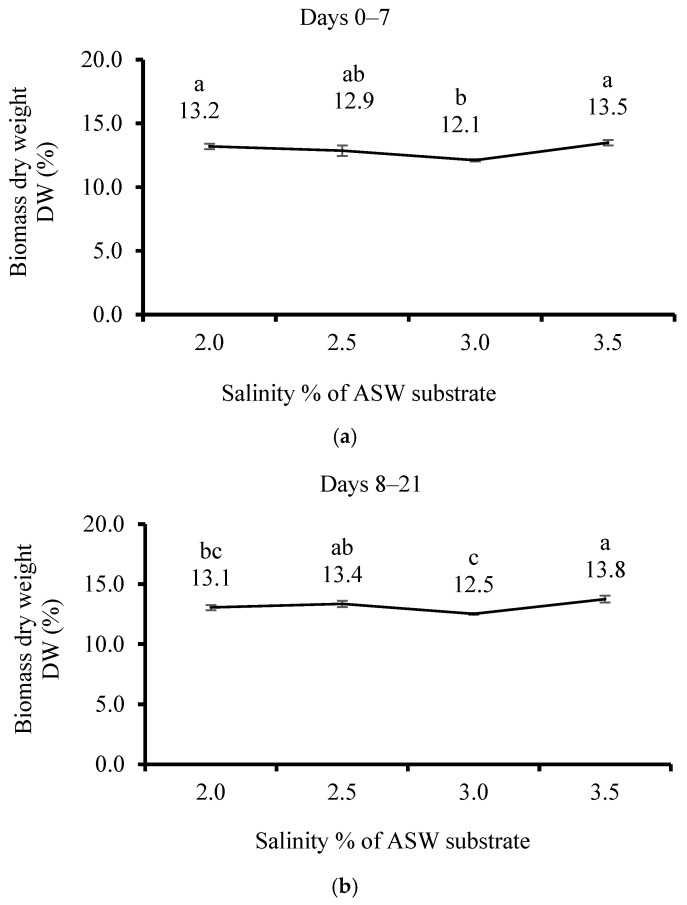
The effect of salinity in the artificial seawater (ASW) substrate of *Ulva lactuca* cultivated in a sleeve system outdoors in a plastic house on the biomass dry weight (DW, % from wet weight, mean ± SD, *n* = 3) when measured (**a**) after 7 days and (**b**) after 21 days. Different letters above the values indicate significant differences as determined by the one-way ANOVA Tukey HSD test (*p* < 0.05).

The DW results of *U. lactuca* cultivated under 3.0% salinity using ASW in plastic sleeves (Figure 3a) show the lowest DW (12.1%) and the highest at 3.5 and 2.0% salinity (13.5 and 13.2%, respectively). The DW biomass after 21 cultivation days (Figure 3b) increased significantly with increasing salinity (13.1, 13.4, 12.5, and 13.8% for a salinity of 2.0, 2.5, 3.0, and 3.5%, respectively).

**Figure 4:**
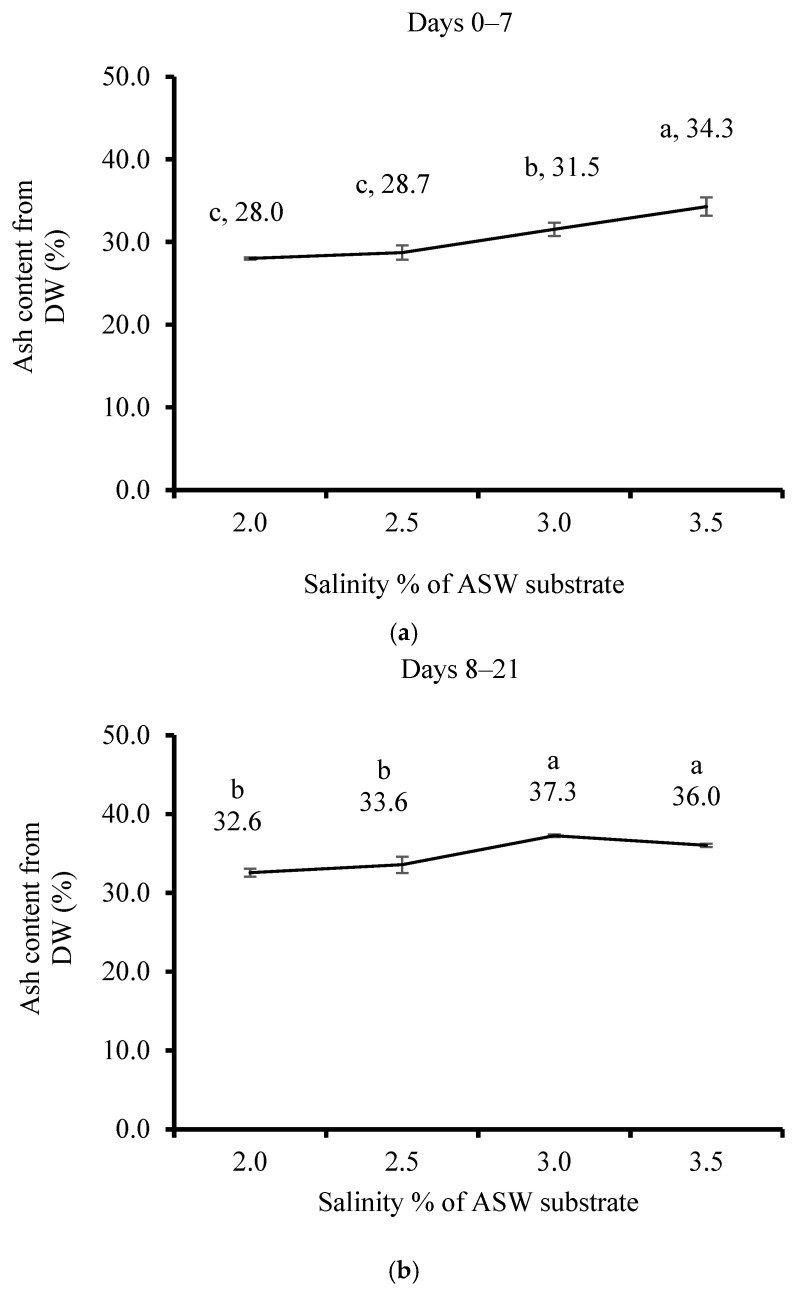
The effect of salinity in the artificial seawater (ASW) substrate of *Ulva lactuca* cultivated in a sleeve system outdoors in a plastic house on the ash content (AC, % from DW, mean ± SD, *n* = 3) when measured (**a**) after 7 days (0–7) and (**b**) 14 days (8–21). Different letters above the values indicate significant differences as determined by the one-way ANOVA Tukey HSD test (*p* < 0.05).

The AC after 7 days (from 28.2 to 34.3%) compared to 21 days of cultivation (32.6 to 37.3%) increased significantly correlated to the ASW salinity increase (Figure 4a,b).

The nitrogen content (N%) in dry biomass after 7 days (Table S1) reached a higher level at a salinity of 2.0 and 3.5% (3.2 ± 0.1 and 3.3 ± 0.1, respectively) compared with the 2.5 and 3.0% treatments (2.9 ± 0.1 and 2.7 ± 0.0, respectively). On the other hand, insignificant differences in N% were found in all treatments after 21 days. The content of carbon (C%), sulfur (S%), and the C/N and S/N ratios had no unanimous trends.

The content of C16:0 (the major SFA) after 21 days of cultivation increased with salinity increments from 45.9 ± 0.6 to 83.5 ± 0.1 (Table 1). The *n-3* FA content (18:4, 18:3, and 16:3 FA) decreased significantly (from 24.8 ± 0.4 to 5.4 ± 0.0) when the salinity increased after 21 days of cultivation. However, these trends were unclear after 7 days. The PUFA content (*n-3* and *n-6*) (Table 1) decreased with increased salinity (from 38.9 ± 0.7 to 6.6 ± 0.1) during 8–21 days of cultivation.

### 3.3. Effect of Temperature

**Figure 5:**
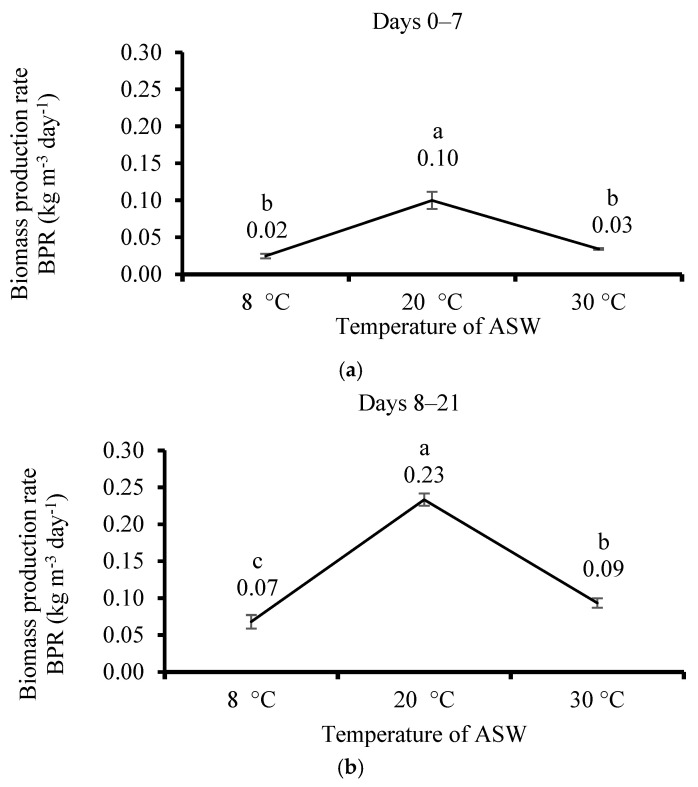

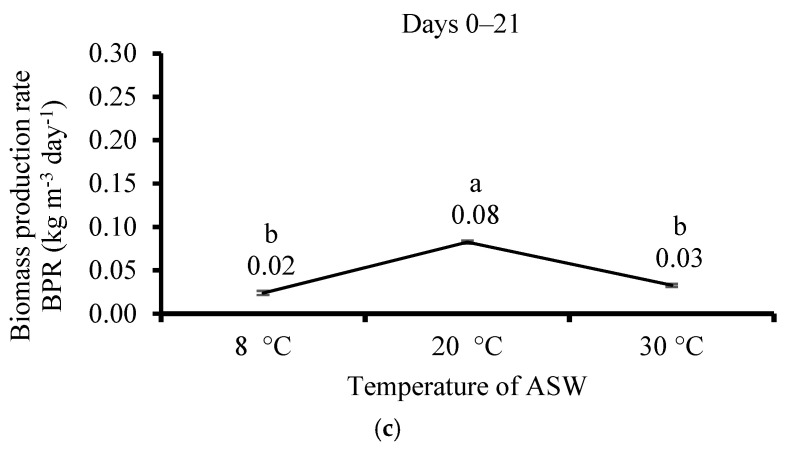
The effect of artificial seawater (ASW) substrate temperature on the cultivation of *Ulva lactuca* on the biomass production rate (BPR, kg m^−3^ day^−1^, mean ± SD, *n* = 3) when measured (**a**) after 7 days (0–7), (**b**) after 21 days (8–21), and (**c**) after the whole 21 days (0–21). The trial was conducted in a flask system placed within three controlled temperature baths set at 8, 20, and 30 °C in outdoors in a plastic house at the Triangle Research and Development (TDRC) site (North Israel) under natural sunlight, with an intensity of 400–800 µmol m^−2^ s^−1^, a 12/12 h photoperiod, and an ASW pH maintained at 8.2. Different letters above the values indicate significant differences according to the one-way ANOVA Tukey HSD test (*p* < 0.05).

After 7 and 21 days of cultivation at different temperatures (8, 20, and 30 °C), the thali of *U. lactuca* grew well but did not change their shape and color. The fresh BPR after 7 days of cultivation showed significant differences between the different temperatures: 20 °C is the highest (0.1), followed by 8 °C and 30 °C (0.02 and 0.03 kg m^−3^ day^−1^, respectively) (Figure 5a). The same trend occurred after 21 days (Figure 5b) and for the whole period (0–21 days) (Figure 5c). The BPR after 21 days was the highest at 20 °C and the minimal at 30 °C and 8 °C (0.08, 0.02 and 0.03 kg m^−3^ day^−1^, accordingly).

**Figure 6:**
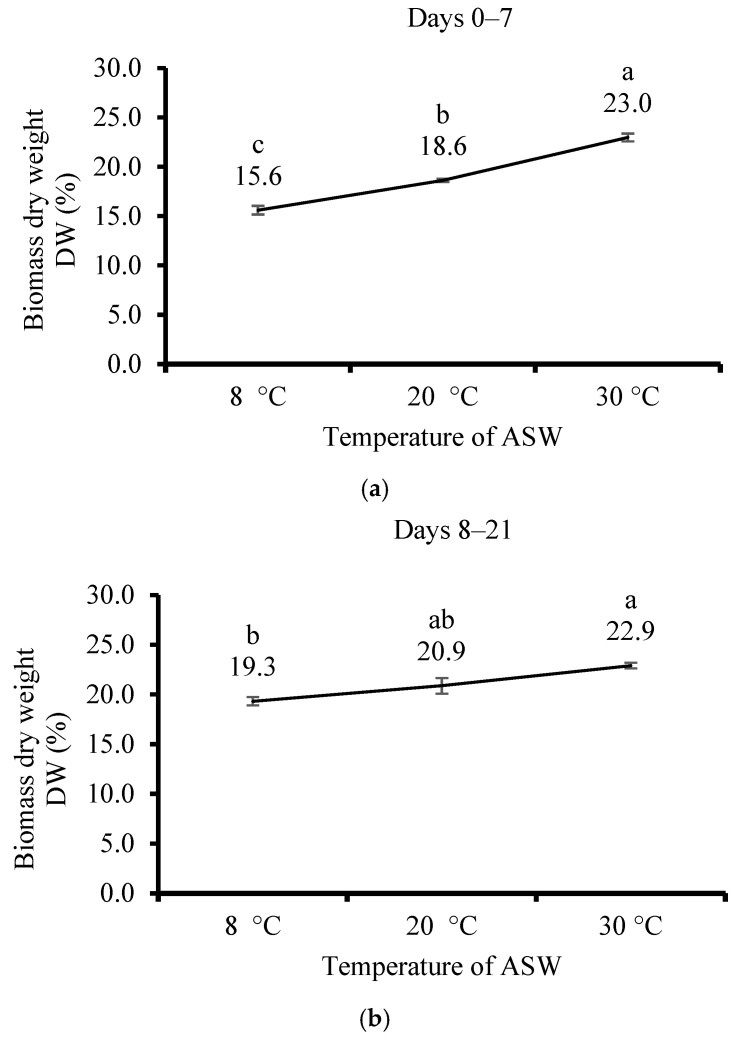
The effect of artificial seawater (ASW) substrate temperature on the cultivation of *Ulva lactuca* when measured (**a**) after 7 days and (**b**) after 21 days. The experiment was assessed in a flask system placed within three controlled temperature baths set to 8, 20, and 30 °C. The dry biomass weight (DW, % of wet weight, mean ± SD, *n* = 3). Different letters above the values indicate significant differences based on the one-way ANOVA Tukey HSD test (*p* < 0.05).

DW mass of *U. lactuca* seaweed cultivated at 8 °C using ASW in flasks was the lowest (23.0%), followed by 20 °C (18.6%). The highest was at 8 °C (15.6%) after 7 days (Figure 6a). The same trend occurred after 21 days (Figure 6b) (from 19.3 to 22.9% for temperatures 8 and 30 °C, respectively).

**Figure 7:**
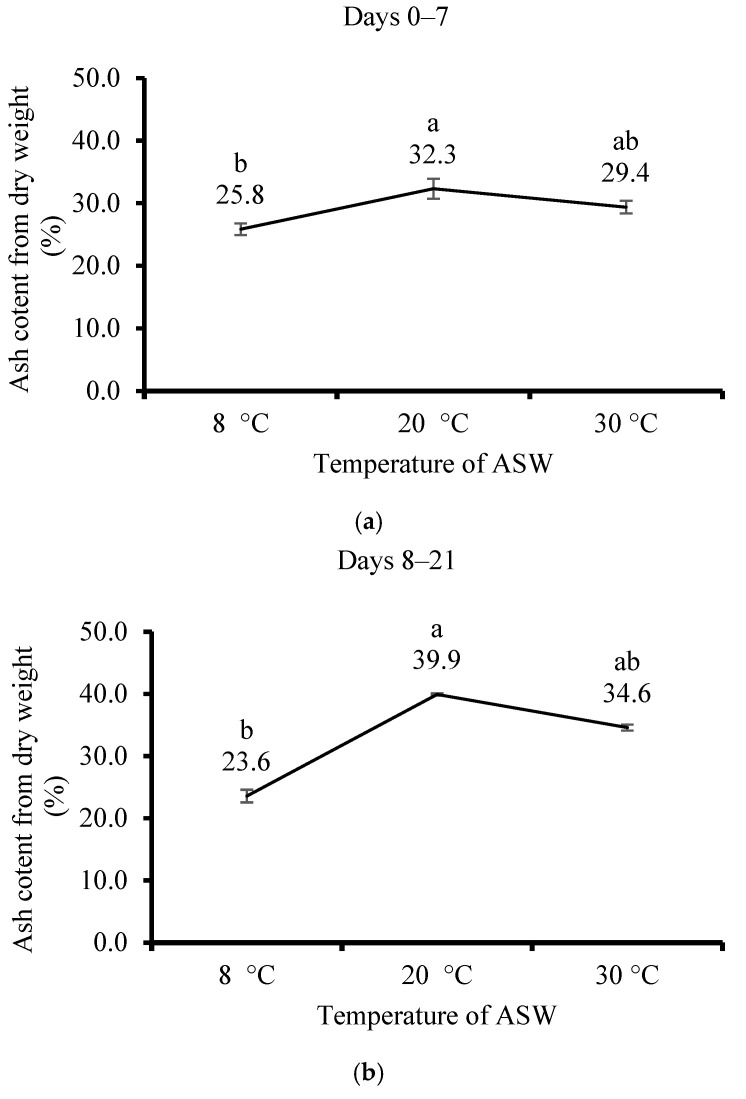
The effect of artificial seawater (ASW) temperature on the cultivation of *Ulva lactuca* on ash content (AC, % of DW, mean ± SD, *n* = 3) when evaluated (**a**) after 7 days and (**b**) after 21 days. Different letters above the values indicate significant differences as determined by the one-way ANOVA Tukey HSD test (*p* < 0.05).

The highest AC value was at 20 °C after 7 days (32.3%) and 21 days (39.9%) of cultivation, compared with 8 °C (25.8 and 23.6% after 7 and 21 days, respectively) and 30 °C (29.4 and 34.6% after 7 and 21 days, respectively) (Figure 7a,b).

Nitrogen content and carbon content in DW after 7 days (Table S2) reached a high level at 8 °C (3.5 ± 0.3, 29.6 ± 0.3, respectively) compared with 20 °C (1.9 ± 0.1, 25.2 ± 0.1, respectively) and at 30 °C (2.2 ± 0.4, 26.3 ± 1.2, 2.0 ± 0.1, and 26.8 ± 0.2, respectively) after 7 and 21 cultivation days. The content of sulfur and the C/N and S/N ratios had no unanimous trends.

The C16:0 content after 7 days increased significantly (from 62.3 ± 2.5 to 77.4 ± 0.5) with temperature increments, but after 21 days, it was insignificant (Table 2). The *n-3* FA (18:4, 18:3, and 16:3) content decreased when the temperature increased after 7 and 21 days (from 7.9 ± 1.0 to 5.5 ± 1.7 and from 17.0 ± 2.0 to 5.7 ± 0.8, respectively), but these trends were insignificant. The content of SFA increased significantly after 7 days when the temperature was increased (from 67.8 ± 4.0 to 81.0 ± 0.8), but after 21 days, these trends were insignificant (from 67.5 ± 1.4 to 77.9 ± 0.7). The content of PUFA after 7 days (Table 2) decreased significantly when the temperature increased (from 25.2 ± 1.5 to 8.5 ± 2.1) but was insignificant after 21 days. The ratio of *n-6/n-3* was increased when the temperature increased to 20 and 30 °C beyond the ideal ratio, supporting human health.

### 3.4. Effect of Nutrients

**Figure 8:**
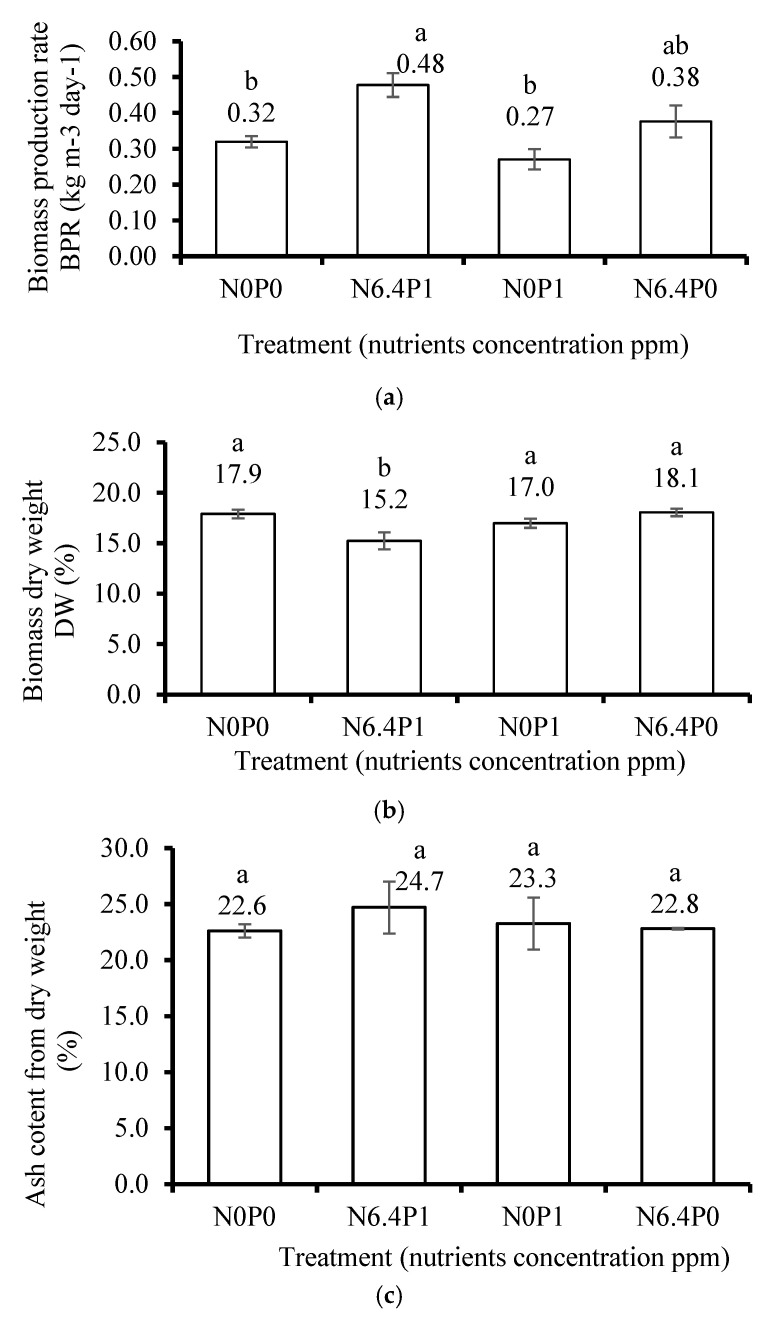
The effect of nutrient (N and P) concentrations in the substrate on (**a**) the biomass production rate (BPR, kg m^−3^ day^−1^, *n* = 3), (**b**) the dry biomass weight (DW, % of fresh weight, mean ± SD, *n* = 3), (**c**) the ash content (AC, % of DW, mean ± SD, *n* = 3). A total of 12 fresh samples were collected of *Ulva lactuca* thalli cultivated in a flask system (3 replicates for each of the 4 treatments) involving different N and P concentrations in the substrate. The experiment was conducted indoors using flasks as photobioreactors at the Triangle Research and Development (TDRC) site. Treatment codes: N0P0 (0 ppm N, 0 ppm P), N6.4P1 (6.4 ppm N, 1 ppm P), N0P1 (0 ppm N, 1 ppm P), and N6.4P0 (6.4 ppm N, 0 ppm P). The cultivation was under photosynthetically active radiation (PAR) from LED lights at an intensity of 100 µmol m^−2^ s^−1^, with a 12/12 h light/dark photoperiod. The harvest occurred after 21 days of cultivation. The artificial seawater (ASW) salinity and pH were maintained at 3.5% and 8.2, respectively, with a constant temperature of 25 °C. Different letters above the bars indicate significant differences as determined by the one-way ANOVA Tukey HSD test (*p* < 0.05).

After 21 days of cultivation in the flasks, the thali of *U. lactuca* did not change their shape and color. The fresh BPR during the 21-day cultivation (Figure 8a) showed significant differences between nutrient levels: N6.4P1 was by far the highest compared with N0P0 and N0P1 (0.48, 0.32, and 0.27 kg m^−3^ day^−1^, accordingly), but it showed insignificant compared with N6.4P0 (0.38 kg m^−3^ day^−1^). The DW mass of *U. lactuca* cultivated using N0P0, N0P1, and N6.4P0 nutrient concentrations in ASW (Figure 8b) was significantly higher than that of the N6.4P1 treatment. The DW of N0P0, N0P1, and N6.4P0 revealed insignificant differences. There were negligible differences in AC between all treatments (Figure 8c). The nitrogen and phosphorus content in the DW of biomass is significantly correlated with the concentration of these elements in the substrate (Table S3). The carbon content in dry biomass after 21 days significantly reached a higher level at the low N concentration treatments using N0P0 and N0P1.

The C16:0 content after 21 days of treatment with N0P0 was significantly higher than that with N6.4P0, but it was insignificant compared with the remaining treatments (Table 3). The *n-3* FA 18:4 content increased insignificantly from 8.9% ± 1.6 to 12.8% ± 0.9 using N0P0, N6.4P1, N0P1, and N6.4P0, respectively. The other *n-3* FA (i.e., 18:3) content increased significantly from 8.9% ± 1.6 to 11.8% ± 0.8 when N0P0 was compared with N6.4P0. The SFA content decreased significantly from N0P0 to N6.4P0 (59.5% ± 3.0 to 50.2% ± 2.5, respectively). The content of PUFA after 21 days (Table 3) increased significantly from 29.1% ± 2.6 to 37.0% ± 2.8 when going from the N6.4P1 to N6.4P0 treatments, respectively. The treatments showed no notable variation in the *n-6* fatty acid content. Thus, the differences in the *n-6/n-3* ratio between treatments show significance and varied from 0.2 ± 0.0 to 0.4 ± 0.1 when going from N0P0 to N6.4P0, respectively.

### 3.5. Effect of Irradiation

**Figure 9:**
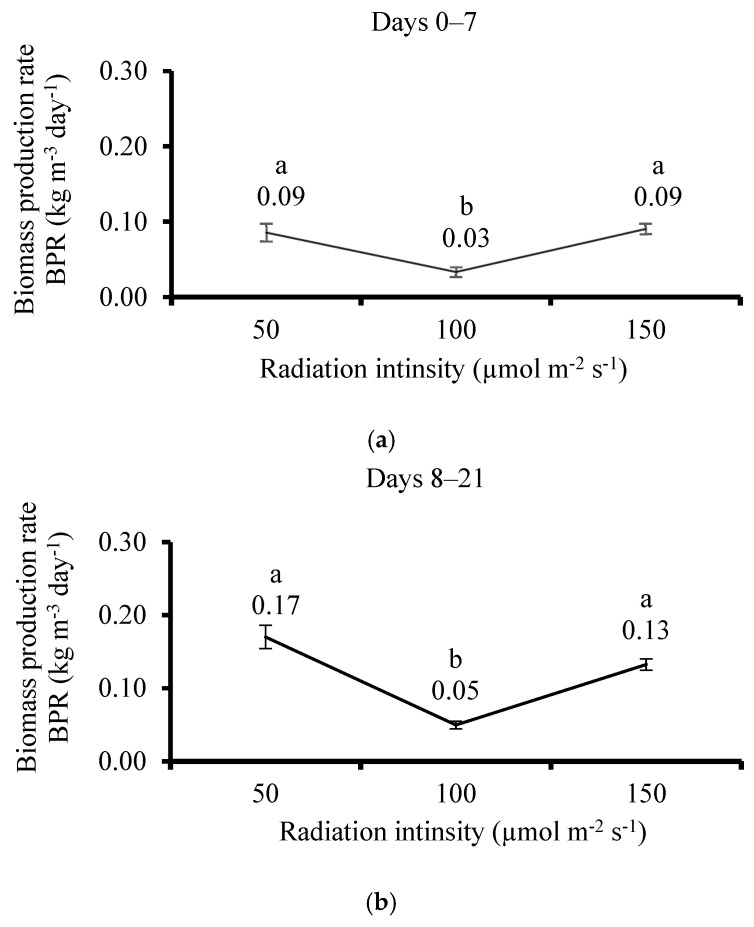
The effect of irradiation of fresh thali of *Ulva lactuca* in flasks system on the biomass production rate (**a**) after 7 days and (**b**) after 21 days (BPR, kg m^−3^ day^−1^, *n* = 3) in seaweed samples used as photobioreactor. Different letters near values above the bars express significant differences using the one-way ANOVA Tukey HSD test (*p* < 0.05).

The thalis of *U. lactuca* did not change their shape and color after 7 or 21 days of cultivation in all treatments. The fresh BPR during the cultivation after 7 and 21 days showed that the 100 µmol m^−2^ s^−1^ level was by far lowest compared with the 50 and 150 µmol m^−2^ s^−1^ levels (Figure 9a,b) (0.03 and 0.09 kg m^−3^ day^−1^ after 7 days; 0.05 and 0.13 kg m^−3^ day^−1^ after 21 days, accordingly). Insignificant differences were detected among the 50 and 150 µmol m^−2^ s^−1^ levels after 7 and 21 cultivation days.

**Figure 10:**
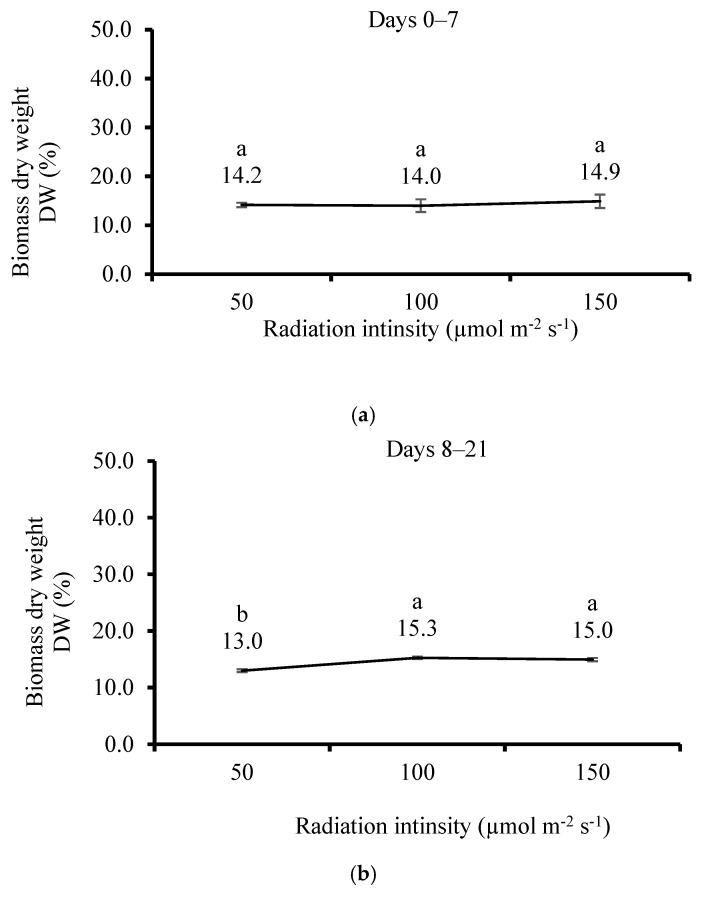
Effect of irradiation on *Ulva lactuca* in flasks system on dry weight (**a**) after 7 days and (**b**) after 21 days (DW, % of fresh weight, mean ± SD, *n* = 3). Different letters near values above the bars express significant differences using the one-way ANOVA Tukey HSD test (*p* < 0.05).

**Figure 11:**
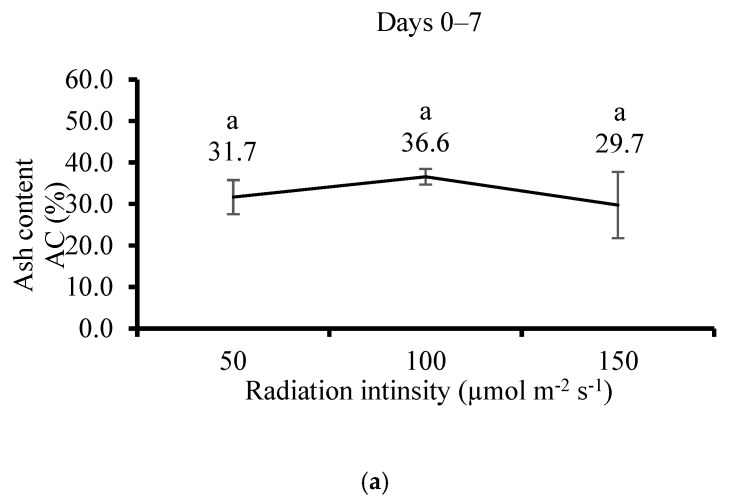

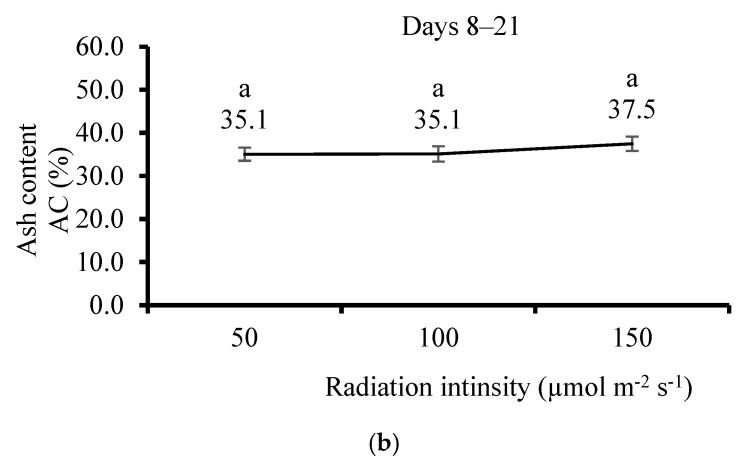
Effect of irradiation on *Ulva lactuca* in flasks system on ash content AC and dry weight DW (**a**) after 7 days and (**b**) after 21 days (AC, % of DW, mean ± SD, *n* = 3). Different letters near values above the bars express significant differences using the one-way ANOVA Tukey HSD test (*p* < 0.05).

The biomass DW of *U. lactuca* significantly increased with the increment of irradiation intensity after 21 days of cultivation, but it was insignificant after 7 days (Figure 10a,b). There were insignificant differences in AC between treatments on both cultivation dates (Figure 11a,b).

The nitrogen, carbon, and sulfur content in DW was insignificantly different in all irradiation treatments after 7 and 21 days (Table S4). Insignificant differences were observed in dry biomass, H%, and the C/N and S/N ratio after 7 and 21 days. The C16:0 content after 21 days of cultivation under 100 µmol m^−2^ s^−1^ treatment was by far the highest among 50 and 150 µmol m^−2^ s^−1^ (63.3% ± 1.4, 56.8 ± 2.0 and 56.3 ± 1.8%, respectively), but an insignificant difference was observed between 50 and 150 µmol m^−2^ s^−1^ (Table 4). The concentration of FA 18:4 (*n-3*) was significantly lower at 100 µmol m^−2^ s^−1^ compared with that of 50 and 150 µmol m^−2^ s^−1^ (6.0 ± 0.3, 9.1 ± 0.9 and 9.9 ± 0.9%, respectively); however, insignificant differences were observed when comparing the 50 and 150 irradiation treatments. The same trend (Table 4) was observed for total *n-3*: the lowest content was with 100 µmol m^−2^ s^−1^ irradiation treatment related with 50 and 150 (14.8 ± 1.0, 20.7 ± 2.1 and 21.9 ± 1.9%, respectively). The content of SFA and the *n-6/n-3* relations of dry biomass treated with 100 µmol m^−2^ s^−1^ were more significant than the 50 and 150 treatments (66.1% ± 0.3, 59.2% ± 2.2, and 57.9% ± 2.0, respectively, for SFA). However, insignificant differences were recorded between 50 and 150 µmol m^−2^ s^−1^. Negligible differences were observed at all treatments regarding the content of *n-6* and MUSFA.

## 4. Discussion

Evaluating the potential of field seaweeds to accumulate higher *n-3* FA content beyond their natural content when exposed to different nutrients and abiotic conditions is crucial for justifying seaweeds as a source of ingredients for the food industry. *U. lactuca* was selected to assess this hypothesis because it naturally has a high lipid content, specifically PUFA and *n-3* content, compared with green, red, and brown seaweeds [53].

These research findings support the hypothesis that when salinity decreases in the ASW substrate, biomass growth decreases. This result aligns with [54], who reported that decreased salinity (1.5 to 3.5%) negatively affects *Ulva* growth and biomass production. However, adding nutrients, mimicking freshwater runoff from events of heavy rains, mitigated this effect, enabling *Ulva* to maintain its bloom potential even at lower salinities [54]. The research of [34] showed reduced growth of other *Ulva* species when salinity was between 1.0 and 3.0%. The maximum growth rate was 2.0%, and the minimum was 4.0%. Various species exhibited distinct nitrate and phosphate absorption rates, with nutrient uptake significantly greater at moderate salinity levels than in the extremes. Salinity also had a notable effect on chlorophyll content, resulting in an efficient quantum yield in *Ulva australis* (formerly *Ulva pertusa*) [55] and *U. lactuca* [56]. This is aligned with the result of this research demonstrating dark green coloring and curling of thali when exposed to 2.0% salinity, which may be related to the low osmotic pressure of the ASW substrate with 2% salinity compared to high osmotic pressure when salinity of 2.5–3.5%. Improved growth at lower salinity levels suggests *Ulva australis* exhibits a higher initial growth rate during the rainy season, typically in late spring and early summer, compared to other periods. Salinity fluctuations may be more significant in triggering *Ulva* blooms in eelgrass habitats than other ecological factors, such as irradiation or temperature [55]. Manipulative cultivation experiments that examined the effect of light (50, 100, and 160 μmol photons m^−2^ s^−1^), temp. (13 and 18 °C), nitrate concentration (<5, 150, and 500 μM), phosphate concentration (<1 and 50 μM), and CO_2_ (200, 400, and 2500 ppm) on the comparative growth rate of *U. fenestrata* (previously *U. lactuca*) indicated that high irradiance (160 μmol photons m^−2^ s^−1^) and low temp. (13 °C) was optimal for growth. However, low irradiance resulted in high FA content, whereas low temperatures negatively impacted FA content, but stimulated the PUFA increase. The addition of nutrients, particularly nitrate, enhanced the FA content, consistent with the findings of this study [45,57]. The different concentrations of nutrients are directly linked to much better biomass production, nutrient uptake, and relative growth rate (RGR) of *U. lactuca* [58]. High irradiance (160 μmol photons m^−2^ s^−1^) and relatively low temp. (13 °C) were optimal for the growth of *U. fenestrata,* but modifying nutrient availability or CO_2_ levels did not enhance the growth rate, likely because the irradiance required for optimal growth of *U. lactuca* is reported to reach saturation at 55 μmol photons m^−2^ s^−1^ [45]. This finding differs from the results of this research, which revealed that a N concentration of 6.4 or 1 ppm significantly increased the BPR. BPR across the treatments shows increments when levels are optimal for *U. lactuca* (low salinity, high or low irradiance, medium temperature, and high nutrient levels). On the other hand, DW% increased when conditions were not favorable to *U. Lactuca*, indicating that stressing conditions increase the DW, which could be explained by high fiber content in cell walls and lower water content [45]. The average ash content level for *U. fenestrata* was about 19 and 20% DW [45], compared with insignificant differences between nutrient concentrations, which varied between 22.6 and 24.7% in this study. The addition of higher nitrate levels and elevated ash matter (22.68% DW) significantly affected the ash level related to macroalgae exposed to moderate nitrate addition (19.05% DW) and high (17.33% DW) nitrate concentrations [45]. High ash content is positively correlated to high BPR and mineral content, which is reached by elevated bioactivity and metabolism in the favorite growing conditions in this research [57].

The proportion of C18:3n3, C16:4n3, C18:4n3, and total *n-3* significantly increased with decreasing irradiance, temperature, and salinity after 21 cultivation days, in agreement with the report of [45]. Compared to C16:0, total SFA increased with increasing salinity, temperature, and irradiation. The low N level in this study increased the *n-3* FA content, which disagrees with the findings of [45]. The PUFA content was also influenced in this study, and it increased with decreased salinity after 21 days of exposure, in agreement with [34]. Temperature is correlated with PUFA content in *Ulva* species: maximum levels were observed in winter and spring (lower temperatures) [30], indicating the potential of *Ulva* for use in human and animal food. Ref. [59] reported that higher antioxidant activity was achieved by lowering the salinity to 1.0% for 10 days. Algal-based aquaculture feed (a blend of macroalgae) is marketed as additives to improve performance and stimulate immunity [37].

Additionally, elevated temperatures, CO_2_, and nitrate levels increased the FA content, suggesting that future ocean environments will enhance the FA production of *Ulva* species [50]. While these results are promising, it is essential to recognize that future ocean conditions might enhance biomass growth and lipid levels yet potentially decrease PUFA content. However, global warming negatively impacts seaweeds’ lipid content by decreasing the PUFA/SFA ratio, the EPA and DHA content, and the *n-6/n-3* ratio [60]. The PUFA supports human health when the ratio of *n-6/n-3* in functional foods is low. A previous study showed that a 0.3 to 0.2 fatty acid ratio lowers the risk of breast, prostate, colon, and renal cancers. In other cases, the *n-3/n-6* fatty acid ratio of 0.5 to 0.3 minimizes inflammation in rheumatoid arthritis patients. A 0.2 ratio, for example, was shown to be effective in asthma patients. In contrast, a fatty acid ratio of 0.1 and lower has been linked to adverse outcomes [22]. In this research, the ratio was at the ideal level under the effect of the various cultivation conditions but increased under 20 and 30 °C beyond the ideal (0.6–1.0) [22]. An *n-6*/*n-3* ratio of <10 is recommended by the World Health Organization (WHO) for nutraceuticals to have the potential to reduce neurological, inflammatory, and cardiovascular disorders; in contrast, the European Nutritional Societies recommend an *n-6/n-3* ratio of <5 [33].

Nutrient availability, particularly nitrogen and phosphorus, also influences PUFA content in seaweeds. The findings of our research are consistent with those of previous studies [37,58,61,62,63,64]. Although this study did not test UV radiation, exploring its role in stress tolerance and antioxidant content is essential. Previous studies have shown that 200 μmol photons m^−2^ s^−1^ is ideal for germling development in *Ulva ohnoi* [65,66], compared with 150 μmol photons m^−2^ s^−1^ in this research. This research revealed that the FA content of the tested seaweed ranged from 0.2 to 1.3% DW and that the highest levels were achieved when the temperature and irradiance were relatively low [67,68]. From a nutritional standpoint, low irradiation strength is recommended to boost lipid and protein levels, but lower temperatures are superior for optimizing protein levels [45]. The results of this research contribute to building a sustainable method of growing seaweeds in aquaculture as an alternative to fish-derived fatty acids and expand the “toolsbox” and manipulation methods and factors to be used by seaweed growers to mimic climate and nutrients situation of favorable seasons to maximize PUFA yield and minimize the *n-6/n-3* ratio to fit the functional food and ingredient products for the food industry.

## 5. Conclusions

This study addressed the potential of green seaweed to serve as a reservoir of bioactive FA compounds, focusing on PUFA. Exposing *U. lactuca* to variable abiotic variables, such as minimal temperature, reduced irradiance, decreased salinity, and elevated phosphorus and nitrogen concentrations, increased the PUFA and *n-3* levels beyond their natural amounts. Future studies should focus on combining the best conditions of irradiation, temperature, and salinity reached in this study to optimize the growth and yield of *n-3*.

## Figures and Tables

**Table 1:** The effect of seawater salt concentration (2, 2.5, 3.0, and 3.5% *w/v*) on the fatty acid (FA) content and the profile of *Ulva lactuca* was assessed after 7 and 21 days of cultivation. The parameters measured included total fatty acids (FAs, % of total fatty acids, mean ± SD, *n* = 3), saturated fatty acids (SFAs, % of total fatty acids), monounsaturated fatty acids (MUFAs, % of total fatty acids), polyunsaturated fatty acids (PUFAs, % of total fatty acids), omega-3 fatty acids (*n-3*, % of total fatty acids), omega-6 fatty acids (*n-6*, % of total fatty acids), and the omega-3/omega-6 ratio (*n-3/n-6*, mean ± SD, *n* = 3). The statistical significance for each parameter is denoted by different letters based on a one-way ANOVA Tukey HSD test. The experiment was conducted using *Ulva lactuca* cultivated in plastic sleeve photobioreactors outdoors at the TRDC site. Twelve fresh samples were collected after 7 days (triplicates for each of the four salinity levels) and another 12 samples after 21 days. Fatty acid content analysis was performed in triplicate (*n* = 3, 3 sleeves).

Fatty Acid	Percentage of Fatty Acid (% *w/w*) of the Total Fatty Acids							
Salinity	2.0% ^8^	2.5%	3.0%	3.5%	2.0%	2.5%	3.0%	3.5%
Cultivation Period	0–7 Days ^9^				8–21 Days			
C14:0	0.8 ± 0.0 a	0.8 ± 0.0 a	0.4 ± 0.0 b	0.1 ± 0.0 c	0.1 ± 0.0 d	0.4 ± 0.0 c	0.8 ± 0.0 b	1.2 ± 0.0 a
C15:0	0.9 ± 0.1 a	0.9 ± 0.1 a	0.2 ± 0.0 b	0.1 ± 0.0 c	0.1 ± 0.0 c	0.2 ± 0.0 b	0.1 ± 0.0 b	0.3 ± 0.0 a
C16:3 *n-6*	0.5 ± 0.0 a	0.5 ± 0.0 a	0.1 ± 0.0 b	0.0 ± 0.0 b	0.2 ± 0.0 a	0.2 ± 0.0 b	0.1 ± 0.0 c	0.1 ± 0.0 d
C16:4 *n-4*	3.3 ± 0.1 a	3.3 ± 0.1 a	2.9 ± 0.0 b	0.0 ± 0.0 c	4.6 ± 0.1 b	3.9 ± 0.1 c	5.6 ± 0.1 a	0.2 ± 0.1 d
C16:3 *n-3*	0.2 ± 0.0 a	0.2 ± 0.0 a	0.2 ± 0.0 a	0.1 ± 0.0 b	0.3 ± 0.0 a	0.3 ± 0.0 a	0.1 ± 0.0 b	0.0 ± 0.0 c
C16:1 *n-7*	1.5 ± 0.1 b	1.5 ± 0.1 b	2.3 ± 0.1 a	0.3 ± 0.0 c	1.6 ± 0.0 b	1.5 ± 0.1 b	2.0 ± 0.0 a	1.0 ± 0.1 c
C18:2 *n-2*	0.2 ± 0.0 a	0.2 ± 0.0 a	0.2 ± 0.0 b	0.1 ± 0.0 c	0.2 ± 0.0 c	0.3 ± 0.0 b	0.3 ± 0.0 a	0.0 ± 0.0 d
C16:0	68.6 ± 0.4 b	68.6 ± 0.4 b	61.3 ± 0.6 c	77.2 ± 0.3 a	45.9 ± 0.6 d	54.2 ± 0.6 c	61.1 ± 0.6 b	65.4 ± 0.4 a
C17:1 *n-7*	0.3 ± 0.0 a	0.3 ± 0.0 a	0.1 ± 0.0 b	0.0 ± 0.0 c	0.5 ± 0.0 a	0.2 ± 0.0 b	0.1 ± 0.0 c	0.5 ± 0.0 a
C18:3 *n-6*	0.1 ± 0.0 b	0.1 ± 0.0 b	0.2 ± 0.0 a	0.1 ± 0.0 c	0.8 ± 0.0 b	0.9 ± 0.0 a	0.4 ± 0.0 c	0.2 ± 0.0 d
C18:4 *n-3*	6.1 ± 0.2 b	6.1 ± 0.2 b	7.2 ± 0.4 a	3.9 ± 0.1 c	10.1 ± 0.2 a	9.9 ± 0.0 a	8.6 ± 0.1 b	1.3 ± 0.0 c
C18:2 *n-6*	2.6 ± 0.0 b	2.6 ± 0.0 b	3.3 ± 0.2 a	2.2 ± 0.1 c	7.2 ± 0.2 a	7.2 ± 0.1 a	4.3 ± 0.2 b	0.3 ± 0.0 c
C18:3 *n-3*	5.0 ± 0.2 b	5.0 ± 0.2 b	9.8 ± 0.1 a	4.8 ± 0.1 b	14.5 ± 0.2 a	11.7 ± 0.3 b	7.8 ± 0.1 c	4.0 ± 0.0 d
C18:1 *n-7*	7.7 ± 0.2 b	7.7 ± 0.2 b	9.4 ± 0.2 a	7.6 ± 0.1 b	8.5 ± 0.1 a	6.4 ± 0.1 b	6.9 ± 0.2 b	8.7 ± 0.1 a
C18:0	1.3 ± 0.0 a	1.3 ± 0.0 a	0.4 ± 0.0 c	0.6 ± 0.0 b	1.4 ± 0.0 a	0.9 ± 0.0 b	0.7 ± 0.0 c	1.4 ± 0.0 a
C20:4 *n-6*	0.0 ± 0.0 b	0.0 ± 0.0 b	0.1 ± 0.0 a	0.1 ± 0.0 a	0.8 ± 0.0 a	0.1 ± 0.0 b	0.0 ± 0.0 c	0.0 ± 0.0 c
C22:4 *n-7*	0.0 ± 0.0 a	0.0 ± 0.0 a	0.0 ± 0.0 a	0.0 ± 0.0 a	0.6 ± 0.0 a	0.1 ± 0.0 b	0.0 ± 0.0 c	0.1 ± 0.0 b
C22:6 *n-3*	0.8 ± 0.0 a	0.8 ± 0.0 a	0.1 ± 0.0 b	0.0 ± 0.0 c	0.0 ± 0.0 b	0.0 ± 0.0 a	0.0 ± 0.0 b	0.0 ± 0.0 ab
C22:0	0.0 ± 0.0 c	0.0 ± 0.0 c	1.9 ± 0.1 b	2.7 ± 0.0 a	2.5 ± 0.0 b	1.8 ± 0.1 c	0.9 ± 0.0 d	15.3 ± 0.4 a
TFA ^1^	100.0 ± 0.0 a	100.0 ± 0.0 a	100.0 ± 0.0 a	99.9 ± 0.0 a	99.8 ± 0.3 a	100.0 ± 0.0 a	100.0 ± 0.0 a	100.0 ± 0.0 a
SFA ^2^	71.6 ± 0.2 b	71.6 ± 0.2 b	64.3 ± 0.6 c	80.6 ± 0.3 a	49.9 ± 0.6 d	57.4 ± 0.5 c	63.7 ± 0.6 b	83.5 ± 0.1 a
MUFA ^3^	9.5 ± 0.2 b	9.5 ± 0.2 b	11.8 ± 0.3 a	8.0 ± 0.1 c	10.6 ± 0.1 a	8.1 ± 0.1 c	9.0 ± 0.2 b	10.2 ± 0.1 a
PUFA ^4^	19.1 ± 0.1 b	19.1 ± 0.1 b	23.8 ± 0.6 a	11.4 ± 0.2 c	38.9 ± 0.7 a	33.8 ± 0.4 b	27.0 ± 0.4 c	6.6 ± 0.1 d
*n-3* ^5^	12.1 ± 0.1 b	12.1 ± 0.1 b	17.2 ± 0.4 a	8.8 ± 0.1 c	24.8 ± 0.4 a	21.9 ± 0.3 b	16.5 ± 0.2 c	5.4 ± 0.0 d
*n-6* ^6^	3.2 ± 0.0 b	3.2 ± 0.0 b	3.7 ± 0.2 a	2.4 ± 0.1 c	9.0 ± 0.2 a	8.3 ± 0.1 b	4.9 ± 0.2 c	0.6 ± 0.0 d
*n-6/n-3* ^7^	0.3 ± 0.0 a	0.3 ± 0.0 a	0.2 ± 0.0 b	0.3 ± 0.0 a	0.4 ± 0.0 a	0.4 ± 0.0 a	0.3 ± 0.0 b	0.1 ± 0.0 c

^1^ Total fatty acid. ^2^ Saturated fatty acids. ^3^ Monounsaturated fatty acids. ^4^ Polyunsaturated fatty acids. ^5^ Omega 3 fatty acids. ^6^ Omega 6 fatty acids. ^7^ Rate. ^8^ Treatment salinity as the salt concentration *w/v*. ^9^ Days of incubation under the treatment temperature. Different letters near the values of the same line (the same days of incubation) express significant differences using a one-way ANOVA Tukey HSD test (*p* < 0.05).

**Table 2:** The effect of substrate temperature (8, 20, or 30 °C) on the fatty acid (FA) content and the profile of *Ulva lactuca* was examined after 7 and 21 days of incubation. The parameters analyzed included the total fatty acids (FA, % of total fatty acids, mean ± SD, *n* = 3), saturated fatty acids (SFA), monounsaturated fatty acids (MUFA), polyunsaturated fatty acids (PUFA), omega-3 fatty acids (*n-3*), omega-6 fatty acids (*n-6*), and the omega-3/omega-6 ratio (*n-3/n-6*), all expressed as a percentage of the total fatty acids with their respective statistical significance denoted by different letters. The seaweed samples were cultivated outdoors in flasks functioning as photobioreactors at the TRDC site. A total of 9 fresh samples were collected after 7 days (triplicates for each of the three temperature levels) and another 9 samples after 21 days. Fatty acid content was analyzed in triplicate (*n* = 3, 3 flasks).

Fatty Acid	Percentage of Fatty Acid (%) from the Total Fatty Acids					
Temperature	8 °C ^8^	20 °C	30 °C	8 °C	20 °C	30 °C
Cultivation Period	After 7 Days ^9^			After 21 Days		
C14:0	0.8 ± 0.2 a	0.1 ± 0.2 a	0.5 ± 0.1 a	1.2 ± 0.1 a	1.2 ± 0.1 a	0.7 ± 0.0 a
C15:0	1.1 ± 0.2 a	0.1 ± 1.2 b	0.7 ± 0.0 ab	2.3 ± 0.3 a	0.2 ± 0.3 b	0.4 ± 0.0 b
C16:3 *n-6*	0.3 ± 0.0 a	0.4 ± 0.0 a	0.2 ± 0.0 a	1.1 ± 0.0 a	0.3 ± 0.0 b	0.2 ± 0.0 b
C16:4 *n-4*	0.9 ± 0.0 a	0.1 ± 0.0 a	0.2 ± 0.1 a	1.9 ± 0.1 a	1.1 ± 0.3 a	2.4 ± 0.3 a
C16:3 *n-3*	0.2 ± 0.5 b	2.8 ± 0.0 a	0.4 ± 1.0 b	0.4 ± 0.1 a	0.2 ± 0.0 a	0.3 ± 0.0 a
C16:1 *n-7*	1.9 ± 0.1 a	2.1 ± 0.3 a	5.2 ± 0.1 a	6.3 ± 0.1 a	3.7 ± 0.4 a	2.0 ± 0.1 a
C18:2 *n-2*	0.0 ± 0.0 b	3.3 ± 0.0 a	0.8 ± 0.0 b	0.7 ± 0.0 a	0.2 ± 0.0 a	0.0 ± 0.0 a
C16:0	62.3 ± 2.5 b	70.8 ± 0.2 b	77.4 ± 0.5 a	57.0 ± 0.5 a	65.3 ± 2.3 a	73.8 ± 0.6 a
C17:1 *n-7*	0.3 ± 0.0 b	2.0 ± 0.0 a	0.4 ± 0.1 b	1.4 ± 0.0 a	0.3 ± 0.0 a	0.5 ± 0.0 a
C18:3 *n-6*	0.2 ± 0.0 a	0.0 ± 0.0 a	2.2 ± 0.0 a	0.0 ± 0.0 a	0.6 ± 0.1 a	2.0 ± 0.5 a
C18:4 *n-3*	8.2 ± 0.3 a	2.4 ± 0.0 a	1.6 ± 0.3 a	14.5 ± 1.0 a	4.3 ± 0.0 a	2.5 ± 0.4 a
C18:2 *n-6*	3.1 ± 0.4 ab	4.2 ± 0.2 a	0.9 ± 0.2 b	1.3 ± 0.6 b	2.1 ± 0.2 b	4.1 ± 0.1 a
C18:3 *n-3*	9.5 ± 0.2 a	3.8 ± 0.4 a	2.7 ± 0.2 a	2.1 ± 0.7 a	8.3 ± 0.9 a	3.0 ± 0.4 a
C18:1 *n-7*	4.8 ± 1.2 a	3.9 ± 0.2 b	3.1 ± 0.1 c	2.5 ± 0.1 b	6.8 ± 0.2 a	5.1 ± 0.2 a
C18:0	1.5 ± 0.4 a	1.8 ± 0.6 a	1.7 ± 0.1 a	1.8 ± 0.1 a	1.7 ± 0.1 a	1.1 ± 0.0 a
C20:4 *n-6*	2.5 ± 0.0 a	0.7 ± 0.0 a	0.1 ± 0.0 a	0.3 ± 0.3 a	0.8 ± 0.0 a	0.2 ± 0.0 a
C22:4 *n-7*	0.2 ± 0.0 a	0.4 ± 0.2 a	0.4 ± 0.0 a	0.1 ± 0.2 a	0.0 ± 0.0 a	0.0 ± 0.0 a
C22:6 *n-3*	0.0 ± 0.0 a	0.4 ± 1.5 a	0.8 ± 0.2 a	0.0 ± 0.2 a	0.0 ± 0.4 ab	0.0 ± 0.0 b
C22:0	2.1 ± 0.7 a	0.7 ± 0.9 b	0.6 ± 0.1 b	5.2 ± 0.3 a	2.7 ± 0.1 a	1.9 ± 0.0 a
TFA ^1^	100.0 ± 6.7 a	100.0 ± 5.9 a	100.0 ± 3.1 a	100.0 ± 4.8 a	100.0 ± 5.3 a	100.0 ± 2.6 a
SFA ^2^	67.8 ± 4.0 b	73.5 ± 3.1 b	81.0 ± 0.8 a	67.5 ± 1.4 a	71.2 ± 2.7 a	77.9 ± 0.7 a
MUFA ^3^	7.0 ± 1.2 a	8.0 ± 0.5 a	8.8 ± 0.3 a	10.2 ± 0.2 a	10.8 ± 0.6 a	7.5 ± 0.3 a
PUFA ^4^	25.2 ± 1.5 a	20.5 ± 2.3 a	8.5 ± 2.1 b	23.7 ± 3.2 a	17.7 ± 1.9 a	13.0 ± 1.2 a
*n-3* ^5^	17.9 ± 1.0 a	9.4 ± 1.9 a	5.5 ± 1.7 a	17.0 ± 2.0 a	12.8 ± 1.3 a	5.7 ± 0.8 a
*n-6* ^6^	6.2 ± 0.4 a	5.3 ± 0.2 a	3.3 ± 0.2 b	2.7 ± 0.9 a	3.7 ± 0.4 a	6.4 ± 0.6 a
*n-6/n-3* ^7^	0.3 ± 0.0 a	0.6 ± 0.0 a	0.6 ± 0.0 a	0.2 ± 0.0 a	0.3 ± 0.0 a	1.1 ± 0.1 a

^1^ Total fatty acid. ^2^ Saturated fatty acid. ^3^ Monounsaturated fatty acids. ^4^ Polyunsaturated fatty acid. ^5^ Omega 3 fatty acids. ^6^ Omega 6 fatty acids. ^7^ Rate. ^8^ Treatment temperature. ^9^ Days of incubation under the treatment temperature. Different letters near the values of the same line (the same days of incubation) express significant differences using a one-way ANOVA Tukey HSD test (*p* < 0.05).

**Table 3:** The effect of nutrient content (*w/v*) in the ASW substrate (nitrogen at 0 or 6.4 ppm N and phosphorus at 0 or 1 ppm, with combinations N0P0, N6.4P0, N0P1, and N6.4P1) on the fatty acid (FA) content and profile of *Ulva lactuca* was assessed after 21 days of incubation. The parameters measured included total fatty acids (FA, % of total fatty acids, mean ± SD, *n* = 3), saturated fatty acids (SFA), monounsaturated fatty acids (MUFA), polyunsaturated fatty acids (PUFA), omega-3 fatty acids (*n-3*), omega-6 fatty acids (*n-6*), and the omega-3/omega-6 ratio (*n-3/n-6*). All values are expressed as the percentages of total fatty acids, with statistical significance denoted by different letters. The seaweed samples were cultivated in lab glass flask photobioreactors at the TRDC site under photosynthetically active radiation (PAR) from LED bars at an intensity of 100 µmol m^2^ s^−1^ and with a 12/12 h light/dark photoperiod. The ASW salinity and pH were maintained at 3.5% and 8.2, respectively, with a constant temperature of 25 °C. A total of 12 fresh samples were collected after 21 days (triplicates for each of the four nutrient-level treatments). Fatty acid content was analyzed in triplicate (*n* = 3, 3 flasks).

Fatty Acid	Percentage of Fatty Acid (%) of the Total Fatty Acids by Treatments			
Nutrient	N0P0 ^8^	N6.4P 1	N0P1	N6.4P0
Cultivation Period	0–21 Days ^9^	0–21 Days	0–21 Days	0–21 Days
C15:0	0.3 ± 0.0 a	0.3 ± 0.0 a	0.2 ± 0.0 a	0.3 ± 0.1 a
C16:4 *n-4*	6.5 ± 0.2 b	6.7 ± 0.4 b	7.3 ± 0.5 ab	8.1 ± 0.3 a
C16:2 *n-6*	0.6 ± 0.1 a	0.5 ± 0.2 a	0.4 ± 0.2 a	0.4 ± 0.1 a
C16:1 *n-5*	0.4 ± 0.0 a	0.6 ± 0.1 a	0.8 ± 0.1 a	0.7 ± 0.3 a
C16:1 *n-9*	3.9 ± 0.4 a	4.5 ± 0.0 a	4.0 ± 0.1 a	3.7 ± 0.4 a
C16:0	58.7 ± 3.0 a	56.9 ± 2.8 ab	51.3 ± 0.7 ab	49.1 ± 2.3 b
C18:4 *n-3*	8.9 ± 1.6 a	9.1 ± 1.5 a	11.6 ± 0.6 a	12.8 ± 0.9 a
C18:2 *n-6*	6.2 ± 0.7 a	4.3 ± 0.9 a	4.1 ± 1.2 a	3.9 ± 1.1 a
C18:3 *n-3*	7.2 ± 1.3 c	8.5 ± 0.6 bc	10.6 ± 0.4 ab	11.8 ± 0.8 a
C18:1 *n-7*	6.8 ± 0.3 a	7.9 ± 0.9 a	8.9 ± 1.9 a	8.5 ± 0.3 a
C22:0	0.5 ± 0.0 a	0.8 ± 0.2 a	0.7 ± 0.1 a	0.8 ± 0.1 a
TFA ^1^	100.0 ± 0.0 a	100.0 ± 0.0 a	100.0 ± 0.0 a	100.0 ± 0.0 a
SFA ^2^	59.5 ± 3.0 a	57.9 ± 2.9 ab	52.2 ± 0.7 bc	50.2 ± 2.5 c
MUFA ^3^	11.1 ± 0.5 b	13.0 ± 0.9 ab	13.7 ± 2.1 a	12.8 ± 0.3 ab
PUFA ^4^	29.4 ± 3.2 ab	29.1 ± 2.6 b	34.1 ± 2.5 ab	37.0 ± 2.8 a
*n-3* ^5^	16.1 ± 2.9 b	17.6 ± 1.9 ab	22.2 ± 1.0 ab	24.6 ± 1.7 a
*n-6* ^6^	6.8 ± 0.8 a	4.8 ± 1.0 a	4.6 ± 1.4 a	4.2 ± 1.2 a
*n-6/n-3* ^7^	0.4 ± 0.1 a	0.3 ± 0.1 b	0.2 ± 0.1 b	0.2 ± 0.0 b

^1^ Total fatty acid. ^2^ Saturated fatty acid. ^3^ Monounsaturated fatty acids. ^4^ Polyunsaturated fatty acid. ^5^ Omega 3 fatty acids. ^6^ Omega 6 fatty acids. ^7^ Rate. ^8^ Treatment nutrient concentration in part per million (ppm). ^9^ Days of incubation under the treatment temperature. Different letters near the values of the same line (the same days of incubation) express significant differences using a one-way ANOVA Tukey HSD test (*p* < 0.05).

**Table 4:** The influence of irradiation intensity (50, 100, or 150 µmol m^2^ s^−1^) after 21 days of incubation on fatty acids content and profile (FA, % of the total fatty acids, mean ± SD, *n* = 3, statistical analysis significance letter), saturated fatty acids (SFA, % of the total fatty acids, mean ± SD, *n* = 3, significance letter), monounsaturated fatty acids (MUFA, % of the total fatty acids, mean ± SD, *n* = 3, significance letter), polyunsaturated fatty acids (PUFA, % of the total fatty acids, mean ± SD, *n* = 3, significance letter), omega-3 fatty acids (*n-3*, % of the total fatty acids, mean ± SD, *n* = 3, significance letter), omega-6 fatty acids (*n-6*, % of the total fatty acids, mean ± SD, *n* = 3, significance letter), omega-3/omega-6 ratio (*n-6/n-3*, % of the total fatty acids, mean ± SD, *n* = 3, significance letter), in seaweed samples of *Ulva lactuca* cultivated in a flask photobioreactor located at the TRDC.

Fatty Acid	Percentage of Fatty Acid (%) from the Total Fatty Acids		
Irradiation Intensity	50 µmol m^2^ s^−1 8^	100 µmol m^2^ s^−1^	150 µmol m^2^ s^−1^
Cultivation Period	0–21 Days ^9^	0–21 Days	0–21 Days
C15:0	0.6 ± 0.2 a	0.7 ± 0.2 a	0.6 ± 0.0 a
C16:4 *n-4*	4.9 ± 0.9 a	2.9 ± 0.4 b	5.2 ± 0.3 a
C16:2 *n-6*	0.3 ± 0.0 a	0.2 ± 0.1 a	0.2 ± 0.0 a
C16:1 *n-5*	0.1 ± 0.0 a	0.1 ± 0.0 a	0.1 ± 0.0 a
C16:1 *n-9*	3.3 ± 0.0 a	3.0 ± 0.3 a	3.4 ± 0.2 a
C16:0	56.8 ± 2.0 b	63.3 ± 1.4 a	56.3 ± 1.8 b
C18:4 *n-3*	9.1 ± 0.9 a	6.0 ± 0.3 b	9.9 ± 0.9 a
C18:2 *n-6*	3.5 ± 0.3 a	3.8 ± 0.2 a	3.1 ± 0.4 a
C18:3 *n-3*	11.6 ± 1.2 a	8.8 ± 0.8 a	12.0 ± 1.0 a
C18:1 *n-7*	8.0 ± 0.7 a	9.1 ± 1.5 a	8.0 ± 0.1 a
C22:0	1.8 ± 0.4 a	2.1 ± 0.9 a	1.1 ± 0.2 a
TFA ^1^	100.0 ± 0.0 a	100.0 ± 0.0 a	100.0 ± 0.0 a
SFA ^2^	59.2 ± 2.2 b	66.1 ± 0.3 a	57.9 ± 2.0 b
MUSFA ^3^	11.4 ± 0.7 a	12.2 ± 1.7 a	11.6 ± 0.2 a
PUFA ^4^	29.4 ± 2.7 a	21.7 ± 1.4 b	30.5 ± 2.1 a
*n-3* ^5^	20.7 ± 2.1 a	14.8 ± 1.0 b	21.9 ± 1.9 a
*n-6* ^6^	3.9 ± 0.3 a	4.0 ± 0.1 a	3.3 ± 0.4 a
*n-6/n-3* ^7^	0.2 ± 0.0 b	0.3 ± 0.0 a	0.2 ± 0.0 b

^1^ Total fatty acid. ^2^ Saturated fatty acid. ^3^ Monounsaturated fatty acids. ^4^ Polyunsaturated fatty acid. ^5^ Omega 3 fatty acids. ^6^ Omega 6 fatty acids. ^7^ Rate. ^8^ Treatment of irradiation intensity levels. ^9^ Days of incubation under the treatment temperature. Different letters near the values of the same line (the same days of incubation) express significant differences using a one-way ANOVA Tukey HSD test (*p* < 0.05).

## Data Availability

The original contributions presented in this study are included in the article/Supplementary Material. Further inquiries can be directed to the corresponding author.

## Supplementary Materials

The following supporting information can be downloaded at: https://www.mdpi.com/article/10.3390/life15010057/s1, Figure S1: The light intensities (50, 100, and 150 µmol m^−2^ s^−1^) were provided by an LED lamp with a wavelength spectrum; Figure S2: Samples of *Ulva lactuca* thalli that displayed varying shapes; Table S1: The elemental content of salinity test; Table S2: The elemental content of temperature test; Table S3: The effect of nutrient (N and P) concentrations in the substrate on the elemental content; Table S4: The effect of irradiation on the elemental content.

## Abbreviations

AC	ash content
ASW	artificial seawater
BPR	biomass production rate
DHA	docosapentaenoic acid
DW	dry weight
EPA	eicosapentaenoic acid
FA	fatty acids
HUFA	highly unsaturated fatty acids
MUSFA	monounsaturated fatty acid
*n-3*	omega-3 fatty acid
SFA	saturated fatty acid
PAR	photosynthetic active radiation
PUFA	polyunsaturated fatty acid
TFA	total fatty acid
UV	ultra violet radiation
