# Supplementary material for: Fatty Acid Content and Profile in Ulva lactuca in Response to Exposure to Variable Growth Conditions in Indoor Photobioreactors
Source: Life (Basel). 2025 Jan 6;15(1):57. doi: 10.3390/life15010057 (PMC11766515; doi:10.3390/life15010057)
Supplement: Supplementary file 1 [file life-15-00057-s001.zip › life-3390527-supplementary.pdf]

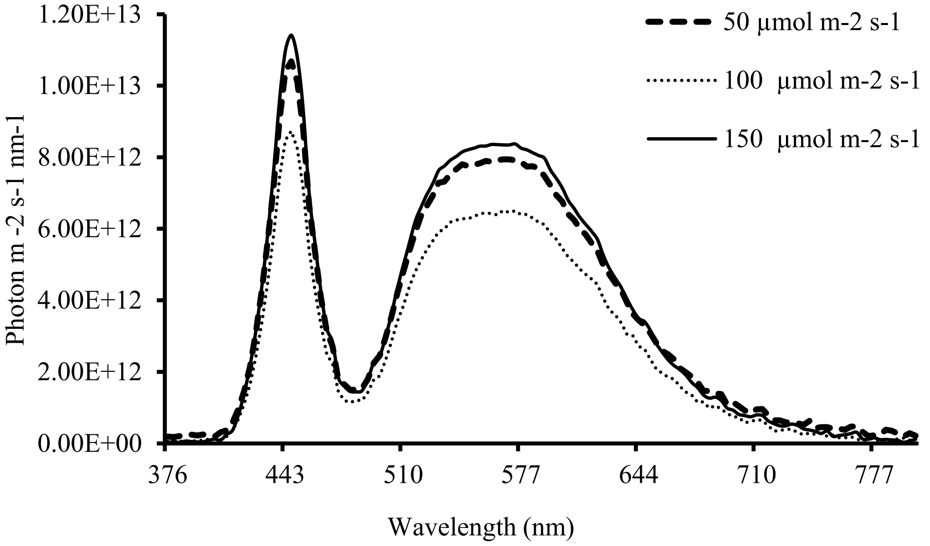

Figure S1. The light intensities (50, 100, and 150 µmol m^{-2} s^{-1}) were provided by an LED lamp with a wavelength spectrum of 376-777 nm were used for the three irradiation treatments in the cultivation of fresh *Ulva lactuca* thalli in a flask system. This photobioreactor setup was located in the laboratory at the TRDC site in northern Israel. A total of 9 fresh samples were collected, with 3 replicates for each of the three light intensity treatments (50, 100, and 150 µmol m^{-2} s^{-1}). Cultivation occurred under photosynthetically active radiation (PAR) from the LED, with a 12/12-hour light/dark photoperiod. The pH and salinity of artificial seawater (ASW) were maintained at 8.2 and 3.5%, respectively, with a constant temperature of 25°C.

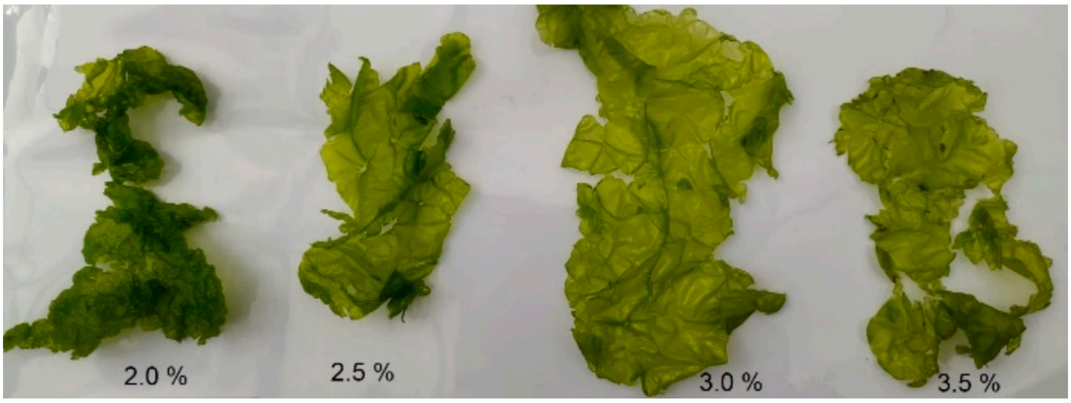

Figure S2. Samples of *Ulva lactuca* thalli that displayed varying shapes (smooth or curled) and colors (dark or pale) at the end of the experiment, conducted with ASW salinity levels of 2.0%, 2.5%, 3.0%, and 3.5%.

Table S1. The elemental content (%) in dry weight (DW) (N%, C%, S%, H% mean ± SD, n = 3), the elemental rate (N/C, S/N mean ± SD, n = 3, statistical analysis significance letters), in seaweed samples of *U. lactuca* cultivated in plastic sleeve photobioreactor located in the TDRC greenhouse (northern Israel). Twelve fresh subsamples were taken after 7 days of cultivation (4 salinity %, red sea salt concentration in ASW treatments, n=3), and twelve fresh samples were taken after 21 days of cultivation (4 salinity treatments, n=3).

Treatment (salinity, %)	Cultivation in the laboratory (days)	Element content (% w/w)*				Elemental ratio	
		N	C	S	H	C/N	S/N
2.0	7	3.2 ± 0.1 a	28.7 ± 0.1 a	4.1 ± 0.1 b	5.2 ± 0.1 a	9.0 ± 0.3 b	3.2 ± 0.1 a
2.5	7	2.9 ± 0.1 b	28.5 ± 0.1 a	4.1 ± 0.1 b	5.3 ± 0.2 a	9.8 ± 0.2 a	2.9 ± 0.1 b
3.0	7	2.7 ± 0.0 b	26.7 ± 0.2 b	4.6 ± 0.2 a	4.8 ± 0.0 b	9.7 ± 0.2 a	2.7 ± 0.0 b
3.5	7	3.3 ± 0.1 a	26.5 ± 0.3 b	3.9 ± 0.1 b	4.7 ± 0.0 b	8.0 ± 0.2 c	3.3 ± 0.1 a
2.0	21	1.9 ± 0.0 a	25.9 ± 0.5 a	4.9 ± 0.1 a	5.0 ± 0.2 a	13.9 ± 0.3 a	4.9 ± 0.1 b
2.5	21	1.5 ± 0.1 a	24.7 ± 0.6 ab	4.9 ± 0.2 a	4.9 ± 0.1 a	17.2 ± 1.4 a	5.0 ± 0.3 a
3.0	21	1.8 ± 0.2 a	24.0 ± 0.9 ab	4.6 ± 0.1 a	4.5 ± 0.1 a	13.7 ± 1.2 a	5.2 ± 0.3 a
3.5	21	1.7 ± 0.1 a	23.5 ± 0.3 b	5.0 ± 0.2 a	4.6 ± 0.1 a	13.5 ± 0.9 a	4.7 ± 0.2 a

* Different litters near the values of the same line express significant differences using a one-way ANOVA Tukey HSD test ($p < 0.05$).

Table S2. The elemental content (%) in dry weight (DW) (N%, C%, S%, H% mean ± SD, *n* = 3, the statistical analysis significance letters), elemental ratio (N/C, S/N mean ± SD, *n* = 3), in seaweed samples of *U. lactuca* cultivated in a flask photobioreactor located at the TDRC site (northern Israel). Nine fresh samples were taken after 7 days of cultivation (3 temperature treatments, n=3), and 9 fresh samples were taken after 21 days of cultivation (3 temperature treatments, n=3).

Treatment (Temperature, ºC)	Cultivation in the laboratory (days)	Element content (%)*				Elemental ratio	
		N	C	S	H	C/N *	S/N
8	7	3.5 ± 0.3 a	29.6 ± 0.3 a	4.0 ± 0.4 a	5.2 ± 0.2 a	8.5 ± 0.9 b	3.5 ± 0.3 a
20	7	1.9 ± 0.1 b	25.2 ± 0.1 b	4.6 ± 0.4 a	4.8 ± 0.2 a	13.3 ± 0.8 a	1.9 ± 0.1 b
30	7	2.2 ± 0.4 ab	26.3 ± 1.2 ab	4.5 ± 0.1 a	4.6 ± 0.1 a	12.0 ± 1.6 ab	2.2 ± 0.4 ab
8	21	4.2 ± 0.0 a	31.0 ± 0.4 a	3.6 ± 0.4 b	5.1 ± 0.0 a	7.4 ± 0.1 b	4.2 ± 0.0 a
20	21	1.7 ± 0.3 b	22.3 ± 1.2 c	5.0 ± 0.5 a	4.4 ± 0.2 a	13.1 ± 1.7 a	1.7 ± 0.3 b
30	21	2.0 ± 0.1 b	26.8 ± 0.2 b	4.0 ± 0.2 ab	4.8 ± 0.2 a	13.4 ± 1.2 a	2.0 ± 0.1 b

* Different litters near values of the same line express significant differences using the one-way ANOVA Tukey HSD test ($p < 0.05$).

Table S3. The effect of nutrient (N and P) concentrations in the substrate on the elemental content (%) in the dry weight (DW) of *Ulva lactuca* thalli cultivated in a flask system was evaluated. The parameters measured included N%, P%, C%, S%, and H% (mean ± SD, n = 3) and elemental ratios (N/C, S/N, P/N, mean ± SD, n = 3). Twelve fresh subsamples were taken after 21 days of cultivation, corresponding to different N and P concentration treatments in the substrate: N0P0 (0 ppm N, 0 ppm P), N6.4P1 (6.4 ppm N, 1 ppm P), N0P1 (0 ppm N, 1 ppm P), and N6.4P0 (6.4 ppm N, 0 ppm P). The experiment used flasks as photobioreactors located in the laboratory at the TDRC. Cultivation was carried out under photosynthetically active radiation (PAR) from LEDs at an intensity of 100 µmol m^{-2} s^{-1}, with a 12/12-hour light/dark photoperiod and a spectrum suitable for plant growth. The ASW pH and salinity were maintained at 8.2 and 3.5%, respectively, with a constant temperature of 25 °C. Different letters near the values indicate significant differences, as determined by a one-way ANOVA Tukey HSD test ($p < 0.05$).

Treatment (nutrient concentration)	Element content (%)**					Elemental ratio		
	N	P	C	S	H	C/N	S/N	P/N
N 0 ppm P 0 ppm*	2.3 ± 0.1 d	3.0 ± 0.3 c	26.5 ± 0.3 a	4.0 ± 0.0 b	2.9 ± 0.1 b	11.8 ± 0.1 a	1.8 ± 0.4 a	1.0 ± 0.1 b
N 6.4 ppm P 1 ppm	4.9 ± 0.0 a	5.5 ± 0.2 a	23.4 ± 0.2 b	5.7 ± 0.4 a	2.5 ± 0.3 b	4.7 ± 0.3 c	1.1 ± 0.1 c	0.5 ± 0.1 c
N 0 ppm P 1 ppm	3.2 ± 0.2 c	4.6 ± 0.1 b	26.2 ± 0.1 a	4.5 ± 0.1 b	3.9 ± 0.1 a	8.3 ± 0.1 b	1.4 ± 0.4 b	0.8 ± 0.1 b
N 6.4 ppm P 0 ppm	4.3 ± 0.0 b	3.0 ± 0.2 c	23.7 ± 0.2 b	4.4 ± 0.2 b	3.8 ± 0.3 a	5.5 ± 0.3 c	1.0 ± 0.1 c	1.3 ± 0.0 a

* Part per million. ** Different litters near values of the same column express significant differences using the one-way ANOVA Tukey HSD test ($p < 0.05$).

Table S4. The elemental content (%) in dry weight (DW) (N%, C%, S%, H% mean ± SD, *n* = 3), the elemental rate (N/C, S/N mean ± SD, *n* = 3, statistical analysis significance letters), in seaweed samples of *U. lactuca* cultivated in flasks in a photobioreactor located in the TDRC lab (northern Israel). Nine fresh subsamples were taken after 7 days of cultivation (3 radiation treatments, n=3) and nine fresh subsamples were taken after 21 days of cultivation (3 radiation treatments, n=3)

Treatments (Irradiation)	Cultivation in Lab (Days)	Element content (%) N %	C %	S %	H %	Elemental ratio C/N ***	S/N
Natural fresh 30 *	0	4.5 ± 0.2 a	36.9 ± 0.3 a	6.0 ± 0.0 a	2.9 ± 0.1 bc	8.3 ± 0.3 a	1.4 ± 0.1 a
Natural fresh 50	0	4.5 ± 0.4 a	37.0 ± 1.6 a	6.0 ± 0.1 a	2.5 ± 0.3 c	8.2 ± 0.5 a	1.3 ± 0.1 a
50 µmole m^{-2} s^{-1} **	7	3.2 ± 0.2 b	25.9 ± 0.5 b	4.5 ± 0.1 b	3.9 ± 0.1 a	8.2 ± 0.4 a	1.4 ± 0.1 a
100 µmole m^{-2} s^{-1}	7	3.1 ± 0.2 b	26.0 ± 1.2 b	4.4 ± 0.2 b	3.8 ± 0.3 a	8.3 ± 0.3 a	1.4 ± 0.1 a
150 µmole m^{-2} s^{-1}	7	3.1 ± 0.1 b	26.2 ± 0.7 b	4.5 ± 0.2 b	4.0 ± 0.1 a	8.6 ± 0.2 a	1.5 ± 0.0 a
50 µmole m^{-2} s^{-1}	21	3.4 ± 0.6 ab	29.6 ± 4.1 ab	4.8 ± 0.7 b	4.4 ± 0.5 a	8.7 ± 0.8 a	1.4 ± 0.2 a
100 µmole m^{-2} s^{-1}	21	3.5 ± 0.3 ab	25.9 ± 0.6 b	4.2 ± 0.2 b	3.4 ± 0.4 ab	7.5 ± 0.6 a	1.2 ± 0.1 a
150 µmole m^{-2} s^{-1}	21	2.8 ± 0.4 b	25.7 ± 1.8 b	4.3 ± 0.3 b	3.8 ± 0.1 a	9.2 ± 0.7 a	1.5 ± 0.1 a

* Fresh biomasses start-stock control 30 days old. ** Radiation PAR intensity unit. *** Elemental Ratio. Different letters near the values of the same column express significant differences using a one-way ANOVA Tukey HSD test ($p < 0.05$).
